# Integrated Multi-Omics Maps of Lower-Grade Gliomas

**DOI:** 10.3390/cancers14112797

**Published:** 2022-06-04

**Authors:** Hans Binder, Maria Schmidt, Lydia Hopp, Suren Davitavyan, Arsen Arakelyan, Henry Loeffler-Wirth

**Affiliations:** 1 Interdisciplinary Centre for Bioinformatics (IZBI), University of Leipzig, 04107 Leipzig, Germany; schmidt@izbi.uni-leipzig.de (M.S.); lydia.hopp@gmx.net (L.H.); wirth@izbi.uni-leipzig.de (H.L.-W.); 2 Armenian Bioinformatics Institute (ABI), 0014 Yerevan, Armenia; aarakelyan@sci.am; 3 Research Group of Bioinformatics, Institute of Molecular Biology of the National Academy of Sciences of the Republic of Armenia, 0014 Yerevan, Armenia; davitavyan@rau.am; 4 Institute of Biomedicine and Pharmacy, Russian-Armenian University, 0051 Yerevan, Armenia

**Keywords:** integrative cancer bioinformatics, transcriptome, DNA methylome and copy number variation data, lower grade gliomas, self-organizing maps machine learning, modes of genomics regulation

## Abstract

**Simple Summary:**

Data from multiple omics domains were increasingly generated in large-scale tumour studies to enhance our understanding of molecular mechanisms of cancer. We present an integrated cartography of three omics layers combining the transcriptome, methylome, and genome (copy number variations) into a unique mapping scheme which enabled us to decipher functional links within and between the omics domains. Application to lower grade gliomas reveals distinct networks governed either by methylation or copy number variations, both affecting transcriptomics modes of cell activity. The integrated maps provide an intuitive view on tumour heterogeneity across the omics layers distinguishing, e.g., astrocytoma- and oligodendroglioma-like glioma types. In a wider sense, multi-omics cartography deciphers the effect of different omes on tumour phenotypes and their molecular hallmarks with individual resolution.

**Abstract:**

Multi-omics high-throughput technologies produce data sets which are not restricted to only one but consist of multiple omics modalities, often as patient-matched tumour specimens. The integrative analysis of these omics modalities is essential to obtain a holistic view on the otherwise fragmented information hidden in this data. We present an intuitive method enabling the combined analysis of multi-omics data based on self-organizing maps machine learning. It “portrays” the expression, methylation and copy number variations (CNV) landscapes of each tumour using the same gene-centred coordinate system. It enables the visual evaluation and direct comparison of the different omics layers on a personalized basis. We applied this combined molecular portrayal to lower grade gliomas, a heterogeneous brain tumour entity. It classifies into a series of molecular subtypes defined by genetic key lesions, which associate with large-scale effects on DNA methylation and gene expression, and in final consequence, drive with cell fate decisions towards oligodendroglioma-, astrocytoma- and glioblastoma-like cancer cell lineages with different prognoses. Consensus modes of concerted changes of expression, methylation and CNV are governed by the degree of co-regulation within and between the omics layers. The method is not restricted to the triple-omics data used here. The similarity landscapes reflect partly independent effects of genetic lesions and DNA methylation with consequences for cancer hallmark characteristics such as proliferation, inflammation and blocked differentiation in a subtype specific fashion. It can be extended to integrate other omics features such as genetic mutation, protein expression data as well as extracting prognostic markers.

## 1. Introduction

Multi-omics high-throughput technologies are producing a steeply increasing number of data sets which are not restricted to only one but consist of multiple omics modalities extracted from the same samples, e.g., in patient-matched tumour specimens. Such multi-omics data offer tremendous opportunities for enhancing our molecular understanding of biological systems, particularly regarding different but mutually linked layers of genomics regulation usually subsumed as the basic “omes” genome, epigenome, transcriptome, proteome and metabolome (see [1] for a critical view). The joint, integrative analysis of these omics modalities and the development of appropriate computational methods is essential to obtain a comprehensive overview of the otherwise fragmented information hidden in this data. Bioinformatics challenges are the “big” size of this data (currently up to ten-thousands of samples with ten to hundred thousands of items per sample), their heterogeneity (e.g., the different data types and numerical scales) as well as their complexity owing to the different links and relations between them forming networks within and between the omics layers which govern modes of genomics regulation of cell functions. The bioinformatics challenge must consider, in addition to appropriate analysis and knowledge mining methods, data visualization options in order to enable perception of the mutual interactions between the different omics layers in an intuitive fashion, e.g., by their cartography in a gene-centred state space [2]. In the context of cancer, such an integrative bioinformatics analysis addressed important issues such as the better understanding of molecular mechanisms of cancer genesis, progression, extracting prognostic markers, finding drugs for targeted treatment and ways against treatment resistance. The underlying mechanisms are often driven by a complex interplay between the omes including genetic defects, epigenetics reprogramming, and perturbed transcription factor networks. Practical objectives of computational methods are the description of cancer heterogeneity in terms of subtypes and the extraction of prognostic markers from the different “omes”, e.g., by asking whether single omics modalities or combinations of them are better suited and if so, what modality is “the best” or how to combine them optimally [3].

Cancer is not solely a genetic disease where genetic defects such as mutations and copy number variations affect gene regulation and eventually lead to aberrant cell functioning as reflected by changes of gene or protein expression. Epigenetic alterations represent another important layer of (de-)regulation of gene activity [4]. Aberrant DNA methylation is a hallmark of many cancer types and methylation patterns were successfully used to subtype cancer heterogeneity [5]. DNA methylation subtypes such as CpG-island hyper-methylation phenotypes (CIMP) [6] have been described, for example, in colorectal cancer, glioma, and leukaemia. Transcriptomics defines another layer of genomics regulation specifying cancer heterogeneity in terms of biological functions associating with different cellular programs related to hallmarks of cancer such as increased proliferation, resisting cell death, or replicative immortality [7]. Hence, consideration of genetic aberrations of DNA methylation in parallel to gene expression is considered to be inevitable for understanding tumour heterogeneity. Accordingly, an integrative view is required linking the different omics modalities. Such integrative approaches can be based on correlation measures between the data which allows for extracting lists of genes whose expression is affected by DNA methylation and/or mutations [8,9,10]. In addition, co-clustering [11,12,13], meta-dimensional and multi-staged analyses [14], joint non-negative matrix factorization [15] as well as global network analysis across multiple omics layers [16,17] offer further options for integrative studies of high-dimensional, multi-omics data.

For single-omics analysis, we developed a data “portrayal” method based on machine learning using self-organizing maps (SOM) [18,19,20]. The method visualizes the molecular data landscapes in terms of “individual” maps and enables their evaluation by visual inspection as well as by extensive bioinformatics downstream analysis. The method is available as R-program [19] and as an interactive web tool for processed data [21]. Thus far, we applied SOM portrayal in the gene expression and/or the methylation domains to different cancer entities such as gliomas [22,23,24,25], B-cell lymphomas [26,27,28], colon cancer [29,30], and melanomas [31]. A first step towards an integrative analysis was the mapping of multiple data types using SOM portrayal of histone modifications in the epi-genomes of stem and progenitor cells [32]. Another group presented a SOM framework to conduct integration of large-scale cancer genomics data [33]. For deciphering the interplay between co-expressed and co-methylated genes in high grade gliomas, we recently developed a novel combiSOM portrayal approach which directly combines gene expression and methylation data in a joint machine learning step, and which finally enables the joint cartography of both omics modalities [34].

This combined double-omics SOM portrayal is not restricted to expression and methylation data. It can be extended to integrate other omics features such as genetic mutation or copy number data to consider their effect directly. In this publication, we pursue this way and present a triple-omics SOM portrayal method which combines patient-matched gene expression (Gex), DNA methylation (Dme), and copy number variation (CNV) data. We applied this method to lower-grade gliomas (LGG) by following another way of our previous research, namely the multi-omics characterization of this tumour type. Thus far, it has been performed by separately analysing each of the different omics modalities and linking the results only in the final step [25,35]. Our novel combiSOM portrayal always integrates them from the beginning to obtain a multi-omics map of LGG. In the first part of this paper, we describe the method and illustrate its performance in terms of coupled date landscapes which illustrate mutual interactions between the Gex, Dme and CNV domains making use of the genetic classification of LGG proposed by WHO [36]. In the second part, we apply a finer stratification based on the expression and methylation patterns of LGG [25]. We demonstrate that integral SOM cartography provides an intuitive approach enabling to decipher the effect of different omes on tumour phenotypes, their molecular hallmarks and their possible impact for prognosis.

## 2. Materials and Methods

### 2.1. Gene Expression, Methylation, and Copy Number Data of Gliomas

**Figure 1:**
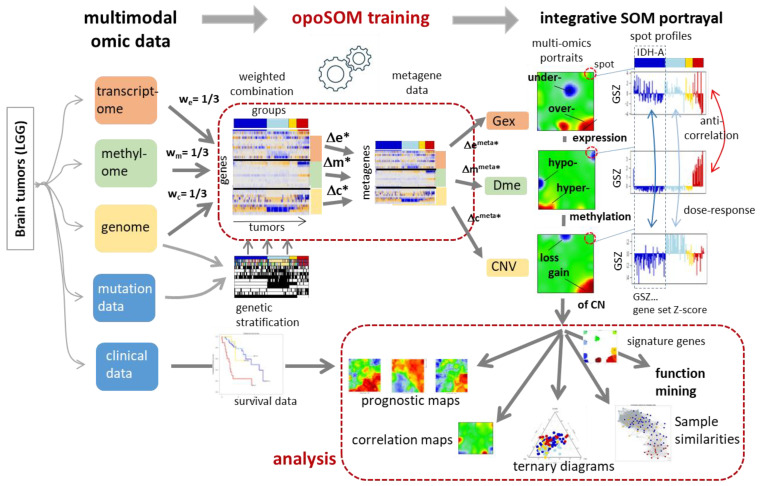
Workflow of integral multi-omics portrayal using combiSOM analysis. Different omics data layers were combined using weight factors and then trained together into one combined self-organizing map (combiSOM). After training, the three layers were decomposed to provide one separate portrait for each of them. Genes are located identically in all three portraits. Comparison of the three profiles reveals virtually anticorrelation between Gex and Dme for the red group and correlation between CNV and Gex for the cyan and blue groups. Downstream analysis considers clinical data to generate prognostic maps, to extract the functional context, and the relatedness between the omes and tumours. Symbols: w_i_ with i = e, m, c denotes the weighting factor for combining the expression, methylation and CNV domains and Δe, Δm and Δc their feature values (see text).

**Figure 2:**
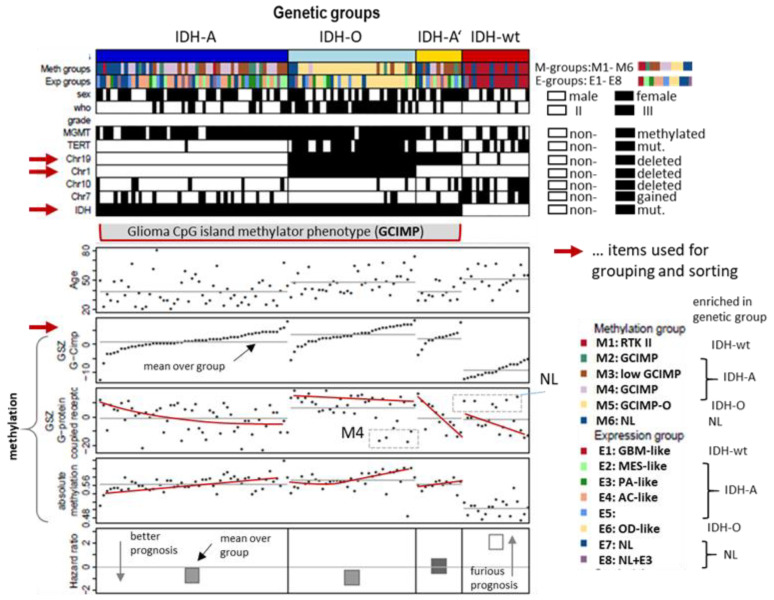
Genetic stratification of LGG, patient, genetic, and methylation characteristics. LGG were classified according to their *IDH* mutation status (*IDH*-mutated tumours were subsumed as GCIMP) and the co-deletion status of Chr.1p/19q, and single deletion of Chr.19q and sorted in each of the groups with increasing GCIMP-methylation score [37]. Selected features such as *TERT* promoter mutation, CNV of Chr.7+ (gains) and Chr.10- (loss), WHO grade (II or III), and prognosis (hazard ratio) differ between the genetic groups. Expression (E1–E8) and methylation (M1–M6) groups as defined in [35] enrich in different genetic groups. The red lines serve as guide for the eye to show trends of GCIMP and G-protein coupled receptor (GPCR) DNA methylation.

We here studied 122 WHO grade II and III adult-type gliomas (lower grade gliomas, LGG) collected from patients which were previously analysed by microarray-based gene expression profiling [22] and array-based DNA methylation profiling (Illumina 450K arrays) [25,35]. The tumours were stratified into genetic groups according to the WHO 2021-classification scheme [36], namely astrocytomas (IDH-A) carrying mutation in the *IDH1* or *IDH2* gene (*IDH*-mut) and no co-deletion of Chr1p and Chr19q (Chr1/Chr19 intact), oligodendrogliomas (IDH-O) with *IDH*-mut and Chr1p/19q codeletions (codel) and *IDH*-wt astrocytoma/glioblastoma-like tumours. In addition, we collected IDH-A gliomas with single Chr19q deletions without Chr1p co-deletions into a separate class IDH-A’. A schematic workflow of the data analysis is provided in Figure 1. LGG subtyping is illustrated in Figure 2.

### 2.2. Preprocessing and Multi-Omics CombiSOM Portrayal

Gene-centric expression (Gex), methylation (Dme), and copy number data (CNV) were preprocessed as described previously [25] and then centralized by subtracting the respective log-mean averaged over all gliomas (see Appendix A, Equation (A1)). The obtained omics scores define profiles given as data vector for each gene with the sample-related values as elements (Appendix A, Equation (A2)). Subsequently, the score values from the different omics modalities were harmonized to make the different numerical scales comparable (Appendix A, Equation (A3)). After centralization and harmonization, we merged the Gex, Dme, and CNV scores of each gene into one combined multi-omics score where the different omics data were combined with different mutual weights, *w_e_*, *w_m_*, *w_c_*, for Gex, Dme, and CNV data, respectively. They were chosen from the interval (0,1) and meet the condition *w_e_* + *w_m_* + *w_c_* = 1 (Figure 1, Appendix A, Equation (A4)).

The combined profile vectors were then clustered into so-called metagene profiles (Appendix A, Equation (A5)) by applying machine learning using Self-Organizing Maps (SOM) using our implementation in oposSOM [19] as a combiSOM version [34]. The method uses an iterative training algorithm to ensure that the metagene profiles optimally cover the omics data space. SOM training arranges the metagenes in a quadratic grid used for visualization of the data in metagene space. We applied a SOM of size 45 × 45 and default parametrization of SOM training. After training, the combined multi-omics scores are back transformed into their original single omics components for visualization and further downstream analysis. Accordingly, each tumour sample studied is characterized by its state in each of the omics domains and visualized by a separate image, namely a Gex, a Dme and a CNV portrait, respectively. Importantly, each metagene and the associated single genes are located at the same position in each of the portraits enabling their direct comparison, e.g., to compare their expression, methylation, and copy number levels. Because SOM training applies to the combined multimodal vectors the topology of the resulting map is governed by the weighting factors, which, in turn, define the degree of couplings between the different omics layers. We here applied equal weights as default setting (*w_e_* = *w_m_* = *w_c_* = 1/3), ensuring balanced couplings between the Gex, Dme and CNV domains in the resulting SOM. Alternatively, one can apply “dominant weights” (e.g., *w_e_* = 0.99, *w_m_* = *w_c_* = 0.0005, for expression dominance) resulting in a single-ome topology of the resulting SOM.

### 2.3. ScoV (Signed Square Root Covariance) Maps and Mean Portraits

Co-variances between the different omics domains can be estimated by calculating the signed square root covariance (ScoV) between their metagene profiles in a pairwise fashion, e.g., between the Gex and Dme, the Gex and CNV; and between the Dme and CNV profiles (Appendix A, Equations (A6) and (A7)). SOM portraits of the three omics domains and of the three combined ScoV were obtained for each sample. For the sample groups, we calculated mean portraits by averaging the respective metagene values over all individual sample portraits of the respective group.

### 2.4. Spot Module Selection and Functional Analysis

Due to the self-organizing properties of the SOM, neighboured metagenes tend to be coloured similarly because of their similar profiles. In consequence, the obtained mosaic images show a smooth texture with red and blue spot-like regions referring to clusters of increased or decreased omics scores in the respective tumour. These “spots” represent clusters of co-expressed, co-methylated, and co-aberrant genes in the Gex, Dme, and CNV domains, respectively. Genes in the spots were identified by applying a threshold (usually 90% of maximum) to the respective omics score [38].

Function mining was performed using a repository of about 6000 gene sets implemented in oposSOM, which refer to different functional context and which were taken from literature, gene ontology and other sources [19]. The gene set score (GSZ) estimates the normalized mean log expression of the member genes of a set in each of the tumour samples (Appendix A) [39]. For Dme and CNV data, the GSZ score is calculated analogously by substituting the expression value by the respective methylation and CNV values. For ternary diagrams, GSZ scores were transformed into percentages of the modality values (Appendix A). Diversity analysis in sample and gene state space as well as function mining by means of gene set analysis was performed using the standard options provided by oposSOM [19,40] (Appendix A).

## 3. Results

### 3.1. Genetic Stratification of LGG

The LGG cases under study were classified into four groups based on genetic characteristics agreeing with WHO 2021 stratification of gliomas [36] (Figure 2): The *IDH*-wt group collects unmutated (wild type) *IDH1* and/or *IDH2* (*IDH*) gliomas. LGG carrying *IDH*-mutations split into the IDH-O (oligodendroglioma) group with co-deletions at Chromosome1p and Chr.19q (Chr1/19codel), the IDH-A and IDH-A’ (*IDH*-mut astrocytomas) groups without Chr1/19codel where IDH-A’ in contrast to IDH-A carries a deletion at Chr.19q. Combined gains of Chr.7 (Chr7+) and losses of Chr.10 (Chr10-) constitute a characteristic of WHO grade IV glioblastomas (GBM). These tumours accumulate in *IDH*-wt LGG (12 out of 19 cases, 63%). In *IDH*-mut, LGG Chr7+ and Chr10- appear mostly uncombined in IDH-A and IDH-A’ where the number of tumours with Chr7+ exceed that with Chr10- in IDH-A (16 versus 6 out of 54 cases, 30% versus 11%). IDH-O LGG are characterized by a high amount of *TERT* promoter methylation. The tumours in each LGG group were ranked with increasing score of the CpG-Island Phenotype (GCIMP)-signature taken from [37] (Figure 2). The mean methylation values averaged over the tumours of each group increase in the order IDH-wt< IDH-A/A’< IDH-O. The overall methylation follows that of the GCIMP signature, showing that GCIMP characterizes the hypermethylation of *IDH*-mut LGG. GCIMP LGG have better prognosis than *IDH*-wt in terms of the hazard ratio (HR), whereas the prognosis of IDH-O is slightly better than that of IDH-A/A’. Group averaged methylation of G-protein receptors (GPCR) shows similar relations between the groups as the GCIMP signature. The GCIMP and GPCR methylation of the tumours in each group however show different slopes (red curves)

We previously classified LGG into eight expression subtypes E1–E8 and six methylation subtypes M1–M6 based on their expression and methylation characteristics where both omics domains were considered independently [25,35]. The E- and M-subtypes accumulate to different degrees in the genetic groups, namely E1 and M1 in IDH-wt, E6 and M5 in IDH-O, and E2–E4 and M2–M4 in IDH-A/A’ (see the colour bars in Figure 2). LGG of the E7, E8, and M6 subtypes are subsumed as neuronal (NL) tumours constituting samples of reduced tumour cell content [25]. NL-type LGG distributes over all genetic groups and partly reflect molecular characteristics of a healthy brain [35]. The present study applies an integrative multi-omics approach to this glioma data in order to illustrate the performance of combined SOM portrayal of omics landscapes in an integrative analysis setting.

### 3.2. Transcriptome, Methylome, and Genome Similarity Patterns of LGG Are Different

**Figure 3:**
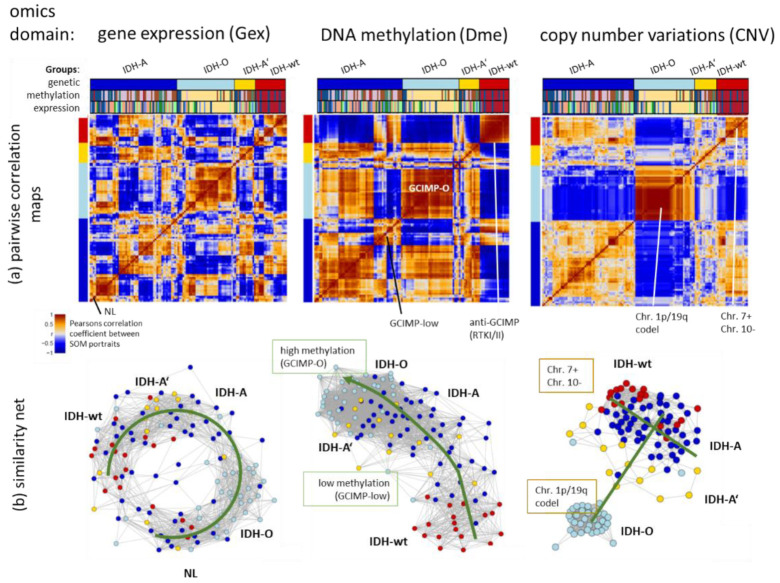
Similarity analysis of LGG using Gex, Dme and CNV data using pairwise similarity provides heatmaps of the SOM portraits (part (**a**)) and similarity network presentation (part (**b**)) (each circle indicates one tumour). CNV shows the clearest separation between genetic groups mainly along two axes referring to the Chr. 1p/19q codeletion and Chr. 7 + status, followed by Dme distributing the tumours along one axis according to their methylation level. Gex produces more diverse, multidimensional patterns resembling a closed circular net with strong intermixing of IDH-A, IDH-A and IDH-wt tumours. For methods description, see Appendix B.

**Figure A1:**
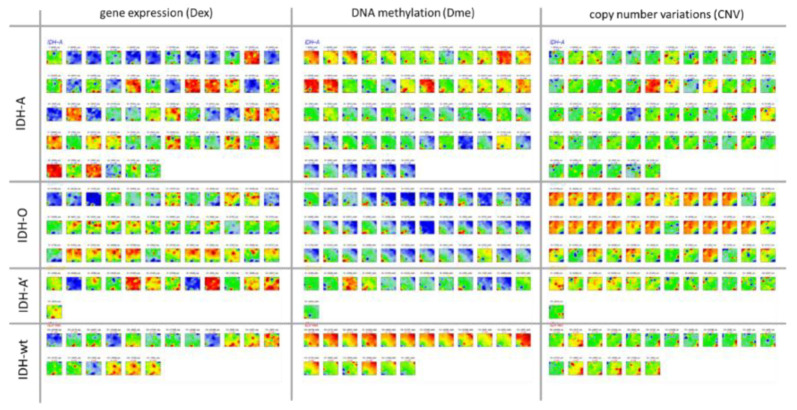
Gallery of single (personalized) tumour portraits in the different omics domains. Mean group portraits are shown in Figure 4a.

Next, we processed integral combiSOM portrayal using gene expression (Gex), methylation (Dme), and CNV data as described in the Methods section. It generated one combiSOM per tumour with one image for each of the three omics realms (Appendix B, Figure A1). Pairwise correlation heatmaps between these portraits characterize the co-variance landscapes of the three omics data (Figure 3a). The maroon-coloured squares along the diagonals mark clusters of tumours with correlated molecular portraits in each of the heatmaps. They mostly agree with the genetic groups, thus reflecting their associations with the Gex, Dme and CNV landscapes. Substructures within the correlation clusters of the genetic groups are indicative of a fine structure of tumour heterogeneity previously resolved in terms of eight expression (E1–E8) and six methylation (M1–M6) glioma subtypes [25,35]. For example, subtypes E7–E8 and M6 can be related to neuronal (NL)-tumours which distribute over all genetic groups but show distinct features differing from other IDH-wt, IDH-A and IDH-O LGG due to their low tumour cell content. Subtypes M2 and E3 induce other sub-patterns in the genetic IDH-A group because of their decreased methylation level forming a separate GCIMP-low methylator type [35]. The Dme heatmap reveals another GCIMP-O methylator type of IDH-O tumours differing from the GCIMP patterns of IDH-A gliomas [35]. The detailed portraits of the E- and M-subtypes in the combiSOM are discussed below.

Network views as implemented in oposSOM [19] visualize topological properties of the sample similarity landscapes: The Dme-network topology is virtually one-dimensional, pointing from low methylation in IDH-wt tumours towards highest methylation in GCIMP-O seen in IDH-O type tumours, with IDH-A and GCIMP-low LGG in between, forming a transition range between low and high methylated gliomas (Figure 3b). The CNV network overall resembles a T-like structure which distributes along two major axes, one spanned by the Chr.1/19 codel versus -intact status and the other which is governed, first of all, by Chr7+ and Chr10- CNVs. The Gex network reflects a more complex, donut-like internal structure with similarity connections between IDH-A/A’ and IDH-wt LGG and separated clouds formed by IDH-O and NL tumour. The three different omics similarity nets clearly separate the IDH-O LGG from the other types, which reflect specific properties of Gex, Dme and CNV data. The other IDH-A/A’ and IDH-wt groups partly mix to different degrees because of overlapping molecular features presumably related to their astrocytoma-like characteristics. In summary, the tumour similarity landscapes of the three omics realms reflect the genetic groups but they also show different complexities ranging from more one-dimensional methylation via a two-dimensional CNV towards a more complex Gex topology.

### 3.3. Integrated Portrayal of LGG Reveals Orthogonal Effects of Methylation and CNV

**Figure 4:**
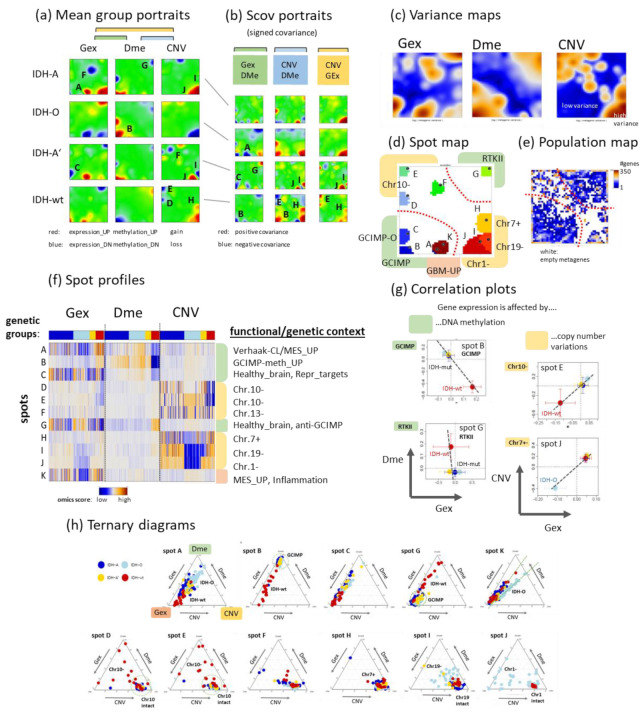
SOM portrayal of the gene expression (Gex), DNA methylation (Dme), and CNV landscapes. (**a**) Mean portraits of the genetic groups indicate characteristic features as red (increased values) or blue (decreased values) spots which are labelled by capital letters. The full gallery of individual portraits is shown in Figure A1. (**b**) ScoV portraits show cross-correlations between pairwise combinations of omics features. Gex and Dme predominantly anti-correlate (blue spots) reflecting repressive effect of DNA promoter methylation on gene activity while CNV and Gex/Dme mostly positively correlate (red) reflecting dose–response relationships. (**c**) Variance maps colour code the gene space for high (maroon colour) to low (blue) variance of the respective omics score. The variance patterns of Dme and CNV distribute along the two perpendicular diagonals, thus reflecting partial independence while the Gex pattern mixes with them. (**d**) The spot map shows the areas of the map with high feature values in any of the group portraits. They are observed predominantly in the Gex (red colour along the frame), Dme (green) or CNV (yellow) domains and are labelled by capital letters A–K in a clockwise direction. (**e**) The population map visualizes the population of metagenes with single genes. In the Dme- and Gex dominated regions (lower left to upper right diagonal), genes are more smoothly distributed while the CNV domain is characterized by an “isolated island”-like distribution of genes. (**f**) Spot profiles in the three omics domains and their functional/genetic context indicate increased (red) and decreased (blue) feature scores. Details are provided in Table A1 (Appendix B) and Supplementary Table S1. (**g**) Correlation plots of genes from selected spots reveal negatively correlated repressive relations between Dme and Gex and positively correlated dose–response relations between CNV and Gex. (**h**) Ternary diagrams of the feature composition in each spot and tumour. Point clouds along the left Gex axis are driven by changing methylation (upper row of diagrams) while the lower row refers to spots governed mostly by CNV Gex dose responses.

The similarity landscapes shown in the previous section describe the state space of the LGG tumours. The SOM portrait, conversely, characterizes the omics state space of the genes chosen as the “atomic” unit of genomics regulation. According to the genetic strata, we generated group averaged Gex, Dme, and CNV portraits (Figure 4a). Red and blue spot-like areas indicate high and low values of the respective omics score, meaning up and down regulated expression, promoter hyper- and hypo-methylation, and copy number gains and losses of the respective genes, respectively. Importantly, our combined portrayal locates each gene at the same position in each of the maps, which enables their direct comparison, e.g., regarding the mutual effects of expression, methylation, and CNV on a certain gene or a group of genes collected in a spot. Overall, the spots refer to combined feature profiles meeting the condition of small mutual *Euclidian* distance between the equally weighted sums of Gex, Dme and CNV features as described in the Methods section (see Appendix B, Equation (A5)). As a result, a spot can collect genes strongly correlated in one of the omics domains but virtually weakly or not correlated in the two other ones, and/or co-correlated in two or all three omics data. For estimating and visualizing the effect of pairwise combinations of different omics features, we generated signed square root covariance (ScoV) maps which colour code negative covariances in blue and positive covariances in maroon (Figure 4b). The Gex versus Dme ScoV maps show mostly blue spots reflecting the repressive mutual effect between gene expression and promoter methylation. In contrast, the Gex versus CNV ScoV maps are dominated by red spots because aberrant copy numbers typically affect expression via a direct dose–response relationship [35].

So-called supporting maps provide additional information about the gene-state space. The variance maps reveal that genes showing large variations of expression, methylation and copy numbers localize in different, only partly overlapping regions of the SOM which reflects partial independence of the different omics domains (Figure 4c). Dme mainly varies along the diagonal axis between the lower left and the right upper corner showing a relatively smooth spot pattern while CNV varies mainly along the other diagonal between the left upper and the right lower corner of the SOM showing a relatively rugged pattern of a larger number of small spots. Variance of Gex more overlaps with that of Dme but partly with that of CNV in distinct spots of high variance due to interactions between the respective omics modalities. The spot map selects distinct areas of high variance, thus defining modules of co-regulated genes in one, two or in all three omics domains using a percentile threshold as described previously [38] (Figure 4d). Overall, eleven major spots were detected which were labelled with capital letters A–K. The population map counts the genes per pixel of the SOM. It reveals that the areas of high Dme variance are occupied by genes continuously distributed across the metagenes while the regions of high CNV variance were occupied more by discontinuously spread “islands” of genes separated by empty (white) metagenes (pixels) in-between (Figure 4e). This difference in gene distribution reflects the continuous feature space of Dme which contrasts the more discontinuous CNV feature space referring to a trinary loss-intact-gain metric.

The spot profiles comprise Gex, Dme and CNV score values across the LGG-samples studied (Figure 4f). They were dominated by alterations of Gex and Dme (green marks), CNV (yellow), or Gex only (red) which associates with the localization of the respective spots in different regions of the SOM (compare with Figure 4d). They can be assigned either to functional gene sets using knowledge mining (mostly Gex dominated spots), to methylation modes (Dme-dominated spots), or copy number aberrations at specific chromosomes (CNV dominated spots). Correlation plots of selected spots show negative correlations between Gex and Dme (spots B and G) and positive correlations between Gex and CNV (spots E and J) (Figure 4g). Ternary diagrams visualize the signal composition in terms of percentages of Gex, Dme and CNV in units GSZ using the spot genes as signature. Accumulation of tumours (dots in the diagrams) along the left Gex axis are indicative of strictly methylation driven gene expression (first row in Figure 4h) while tumours accumulating near the lower CNV axis are governed by a CNV Gex dose–response relation which, however, resembles more an on–off binary switch lacking fine tuning along the CNV axis (second row in Figure 4h). In summary, the combiSOM clusters genes into consensus modes of concerted changes of expression, methylation, and/or CNV and visualizes them in mutually linked landscapes. Their topologies are governed by the degree of co-regulation and the particular interactions between the omics realms studied. Hereby Dme and CNV mainly change along two perpendicular axes reflecting their partial independence while Gex is affected by both of them.

### 3.4. Cartography of Features, Functions, and of Their Prognostic Impact

**Figure 5:**
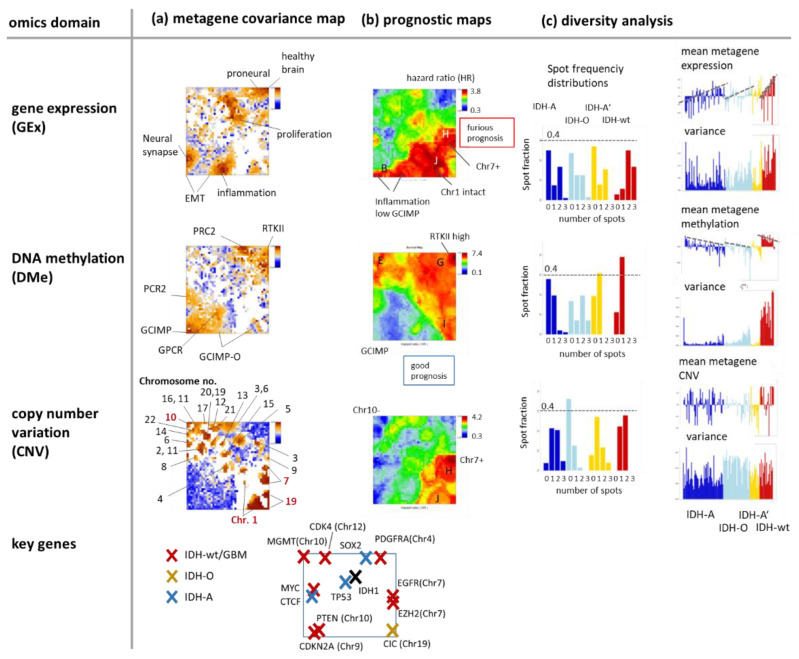
Characteristics of the omics domains: (**a**) The metagene covariance maps colour code the mean covariance between the single-gene profiles and the metagene profiles in each pixel. High covariance values (red) agree with the spot regions. They preferentially associate with cellular programs (Gex), methylation modes (Dme), and chromosome-wise aberrations (CNV). (**b**) The prognostic map colour codes the overall survival (OS) hazard ratio (HR, compared with the mean OS averaged over the whole data set) between maximum (red, furious prognosis) and minimum (blue, good prognosis) HR. HR values are calculated metagene-wise by selecting tumours showing omics scores exceeding one standard deviation in positive direction (i.e., with high values of the score, see [35] for details). (**c**) Diversity analysis includes spot frequency distributions and profiles of the mean omics score and variance per tumour. The row below maps the key genes driving the different genetic subtypes (see Discussion section).

The metagene covariance maps in Figure 5a visualize the mean deviation between the gene and the metagene profiles in each of the pixels [38]. The analysis generates patterns which merge properties of the variance map with that of the population map (compare with Figure 4d,e). Gene set analysis reveals that the highly resolved spot patterns in the different omics maps associate with functional contexts related to gene expression of healthy brain, to proneural, inflammation, and EMT (epithelial-mesenchymal transition) transcriptional signatures in the Gex map, to targets of the polycomb repressive complex 2 (PRC2) related to neural development and to GCIMP, GCIMP-O, GPCR, and RTKII (receptor tyrosine kinase type II methylation subtype) methylation modes in the Dme map and to individual chromosomes in the CNV map where key chromosomal aberrations in gliomas were marked as red chromosome numbers. For example, Chr.19- losses distribute over two neighbouring spots in the right lower corner of the map, one related to Chr.1/19codel in IDH-O and the other one to single Chr.19 deletions in IDH-A’. Glioma key genes were localized across the map (Figure 5a, map below) showing, e.g., an association of *IDH1* and *TP53* with proliferative activity and of *CIC* located at Chr.19 with chromosomal losses of this chromosome in IDH-O and IDH-A’.

The prognostic maps relate Gex, Dme and CNV metagene values across the SOM to the hazard ratio (HR) of patients showing high values of the respective omics score in the respective metagene (Figure 5b, for a detailed description of the method see [35]). For example, the red region in the Gex prognostic map refers to high expression of genes amplified by Chr7 + CNV gains, genes not “suffering” from deletions at Chr1- and genes upregulated in an inflammatory context. These red high-HR areas refer to IDH-wt and partly to IDH-A and GCIMP-low LGG. The blue low-HR areas in this map indicate underexpressed genes in the respective groups. The Dme-prognostic map just shows partly colour-inverted HR-patterns compared with the Gex map where red substitutes blue and vice versa. It can be simply explained by the mostly anticorrelated relation between methylation and expression meaning that high methylation in IDH-wt, e.g., in the right upper corner of the SOM, associates with decreased expression levels. The CNV prognostic map shows similarities with the Gex map due to the correlated dose–response relation between both omics scores. Especially spot H enriching genes of Chr.7 + associates with the most furious prognosis in the CNV and Gex maps where the latter indicates bad prognosis for genes upregulated in the inflammatory context presumably in the tumour microenvironment (spot B) [35]. Notably, the most furious prognosis is predicted by hyper-methylation of spot G (HR > 7) observed specifically in IDH-wt LGG (see the spot profile in Figure 4f) and assigned to the GBM RTKI/II hypermethylation signatures [24,41].

Diversity analysis provided information about the heterogeneity of the molecular patterns in the different omics domains (Figure 5c). The spot frequency distributions count the fraction of tumours with a certain number of spots in their portraits, which, in turn, relates to the molecular heterogeneity of the respective subtypes. Most spots were found in the Gex portraits (up to 3), while Dme portraits show usually only one spot except that of IDH-O LGG with up to three spots. IDH-A shows the most diverse CNV patterns while IDH-O is the less diverse one, mostly related to Chr.1/19 codeletions. The mean metagene expression and methylation profiles (calculated per tumour) confirm their anti-correlated relation.

In summary, covariance summary maps provide highly resolved feature landscapes related to cellular functions, methylation modes, chromosomal aberrations, and key genes. Prognostic information with metagene resolution can be extracted after translating expression, methylation, and CNV metrics into HR-scale with possible impact for marker selection from regions of maximum or minimum HR values. Gene expression landscapes are overall the most diverse ones reflecting largest heterogeneity of cellular processes. Methylation landscapes show lowest heterogeneity presumably because DNA methylation forms a coarser layer of genomics regulation preserving footprints of the respective cell of origin [42]. The CNV landscape is highly diverse where, however, only a few aberrations, such as Chr.1/19 codeletions or Chr.7+ and Chr.10-, seem to be key effectors of LGG heterogeneity considered in the genetic WHO classes. Hence, combined SOM portrayal links the molecular landscapes of expression, methylation, and genetic features with their prognostic impact, which, in turn, associates with key genes, functional and structural signatures of LGG pathogenesis.

### 3.5. Profiling and Mapping Functional Signatures

**Figure 6:**
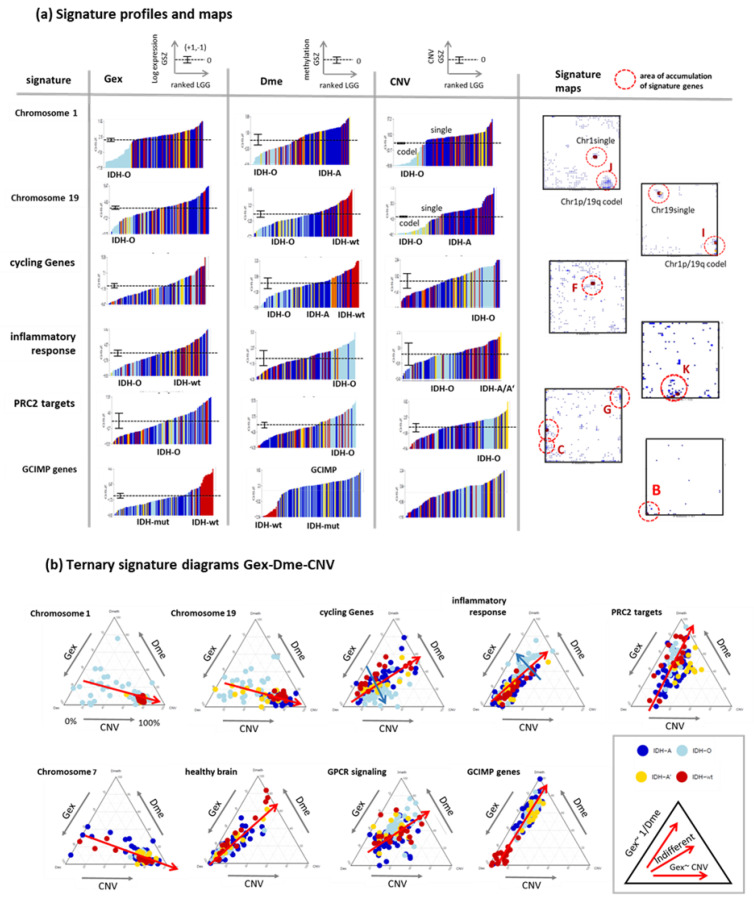
Sets of signature genes were analysed in terms of ranked profiles, maps, and ternary diagrams using the gene set Z score (GSZ) of expression, methylation, and CNV values. (**a**) LGG were ranked with increasing GSZ from left to right. Tumours of different genetic groups accumulate at low or high GSZ values as indicated (LGG-bars are coloured according to their genetic group; see Figure 2 for assignment). The signature maps show the distribution of signature genes in the map. Their accumulation in selected spot areas is indicated by red circles (see Figure 4d). (**b**) Ternary diagrams show mutual dependencies where Gex of the respective set is driven via dose response by CNV (see chromosomal sets), via repression by Dme (see GCIMP set), by combinations of both or none of both (see the legend in the right part). A fine structure spans in direction (blue arrow) perpendicular to the major axis of variation (red arrow). Gene sets were taken from [43,44]. For a larger collection of gene signatures see Figure A2.

**Figure A2:**
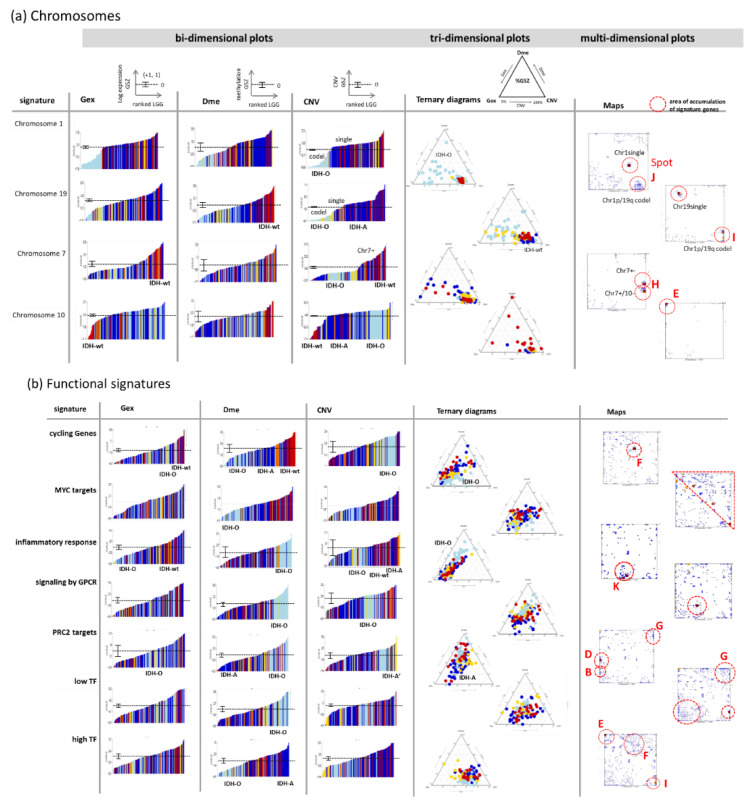

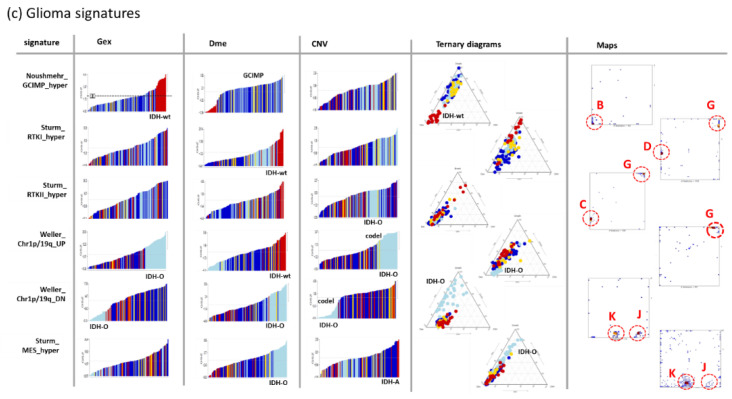
Sets of signature genes were characterized using ranked profiles, ternary diagrams and signature maps. (**a**) Genes from selected chromosomes are typically aberrant in gliomas, (**b**) functional signatures [43,56] and (**c**) signatures differentiating glioma subtypes obtained in previous studies [22,24,37]. In the profiles, the LGG samples were coloured according to the genetic groups IDH-wt (red), IDH-A (blue), IDH-A’ (yellow) and IDH-O (light blue). (**a**) Genes from the key-chromosomal aberration at Chr.1 and 19 (preferentially in IDH-O, partly in IDH-A’) and Chr. 7+ and Chr. 10− (in IDH-wt and, partly, in IDH-A/A’) accumulate in specific areas of the map and reveal orchestrated changes of Gex and CNV values due to dose–response relationships in the ternary diagrams. (**b**) Gene signatures characterizing different biological functions enrich in specific areas of the map. This is indicative of co-regulation of the involved genes which is governed partly by Dme and CNV. For example, genes related to cell cycle activity accumulate in and around spot F and show high expression in IDH-wt and IDH-O. It associates with hypomethylation and a slight copy number gain in IDH-O paralleled by hypomethylation of *MYC*-targets, while cycling genes are hypermethylated in IDH-wt, thus showing the opposite methylation trend. Genes with functions in inflammatory response are downregulated in IDH-O due to their hypermethylation, a pattern, which is partly observed for G-protein coupled receptors (GPCR), thus suggesting their role in this process. Targets of the polycomb repressive complex (PRC2) and so-called low transcription factor activity genes (low TF) are mostly regulated by epigenetic mechanisms in the context of differentiation and development of healthy tissues and cancer [43,56]. Genes from these sets split into two major populations found near opposite corners of the map, namely the lower-left one (Gex_UP in LGG) and the upper right corner (Gex_DN in LGG). This split indicates that the respective genes are affected by an LGG group specific hyper-methylation patterns which activates them either in healthy brain or in brain cancer. Conversely, so-called high-TF genes associate with regulatory modes governed by TF-networks. They accumulate in regions of increased proliferative activity observed for *MYC*-targets. (**c**) Glioma-related signatures were taken from [22,24,37,41]. They show mostly pronounced enrichment in spot areas and clearly associate with group-wise up- and/or down-regulation in the different omics domains. They were adequately extracted from glioma expression data sets. Therefore, these signatures give rise to the spread of the LGG along the Gex axis of the ternary diagram, however with segregation between subtypes, e.g., between IDH-A and IDH-O.

For further knowledge mining in the combined data, we selected gene sets from different categories such as gene ontology biological process (GO BP), signature genes from previous omics studies (mostly Gex), as well as genes located at a certain chromosome. We plotted their ranked profiles, locations of the genes in the SOM as signature maps as well as ternary diagrams in Gex-Dme-CNV space (Figure 6a and Figure A2 for a larger overview). For example, genes from Chr. 1 show low expression in IDH-O tumours due to the loss of copy numbers of Chr. 1, which is associated with slightly decreased methylation levels. These Chr. 1 genes accumulate in and near spot J referring to samples with codeletion together with Chr. 19, but also in a second area which refers to single deletions at Chr. 1 (see red circles in Figure 6a, first raw). Genes from Chr. 19 accumulate in other areas of the map, first of all, in spot I due to frequent codeletion together with Chr.1 in IDH-O as well as in a second spot for LGG with single deletions. CNV dominated alterations of LGG spread roughly along the CNV axis of the ternary diagrams (Figure 6b). GCIMP genes and PRC2-targets accumulate along the Gex axis, which is indicative for Dme-driven expression changes. The distributions of gene signatures related to inflammatory response and of GPCR encoding genes are similar and reflect a strong effect of methylation with subtle differences between IDH-O and IDH-A tumours (see for details [25,35]). Contrarily, cycling genes accumulate around spot F in the map. They form a cloud distributing along the bisectrix between the Gex and CNV axes in the ternary diagram, which indicates small effects of Dme and CNV on Gex, which, in turn, is more governed by transcription factor (TF) network, e.g., via Myc-targets distributing similarly as cycling genes (Figure 6 and Figure A2b). Importantly, most functional signatures show rather continuous distributions despite the systematic differences between the subtypes. In summary, profiling and mapping of gene signatures provide combined information about the mutual effect of gene expression, methylation, and copy number variation on functional modes enabling to evaluate their impact on tumour heterogeneity.

### 3.6. Integrative Portrayal of the LGG Subtype Diversity—Beyond the Genetic Classes

The pairwise correlation maps in Figure 3 reveal a fine structure of glioma heterogeneity not resolved by the genetic groups. Our previous LGG classification resolved this heterogeneity into eight expression (E1–E8) and six methylation (M1- M6) subtypes [25]. The E- and M-subtypes mutually overlap to 70–90% of the tumours which reflects inter-omics regulatory modes between gene expression and DNA methylation modalities, however, the absence of a strong one-to-one relationship [25,35] (see the colour bars above the heatmaps in Figure 2, which assign the LGG to the three different classification schemes). Here we compared the genetic groups with these E- and M groups by means of their combined portraits in the three omics realms in order to identify overlapping and disjunct modes of genomics regulation.

**Figure 7:**
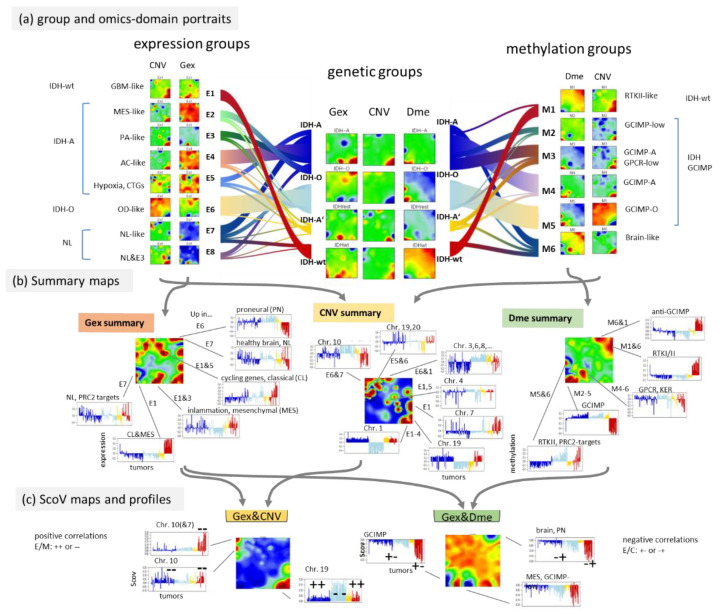
Stratification of genetic groups into expression (E-) and methylation (M-) subtypes defined previously [35]. (**a**) The flow diagrams visualize the distribution of the E- and M groups across the G groups. The group portraits reveal a high diversity of Gex and Dme patterns not fully resolved in the genetic groups (see text). The full gallery of individual tumour portraits is shown in Figure A1. (**b**) The spot summary maps provide an overview of the major spots due to high omics score values (Gex, Dme and CNV) in the respective group portraits. Their omics score profiles were sorted and coloured according to the genetic groups. (**c**) ScoV portraits of the Gex versus-CNV type are dominated by red spots due to positive correlations between gene expression and CNV aberrations while Gex Dme ScoV portraits show predominantly blue spots due to negative correlations between gene expression and DNA methylation. Positive correlations reflect either combined up- (++) or down- (−−), regulation while negative correlations split into up-/down- (+− for Gex_UP and Dme_DN) or down-/up- (−+) combinations.

The flow diagram illustrates the balance of tumours between the genetic groups applied here and the E- and M groups of the previous, independent classifications (Figure 7a). The Gex, Dme and CNV portraits of the IDH-wt genetic group closely resemble those of the E1-expression and M1-methylation subtypes, while the portraits of the IDH-O genetic group are very similar to those of the E6 and M5 subtypes because of the large degree of overlap (see thick flows in Figure 7a). The IDH-A tumours decompose into several substrata (E2–E5 on expression side and M2–M4 on methylation side) where the majority accumulates in the core astrocytoma subtypes E4 and M4 [35]. The NL-like subtypes (E7, E8 and M6) distribute virtually over all genetic groups, which confirms their “technical” origin due to low tumour cell content [25]. Their portraits consequently show partly healthy brain properties not explicitly resolved in the portraits of the genetic groups.

**Figure A3:**
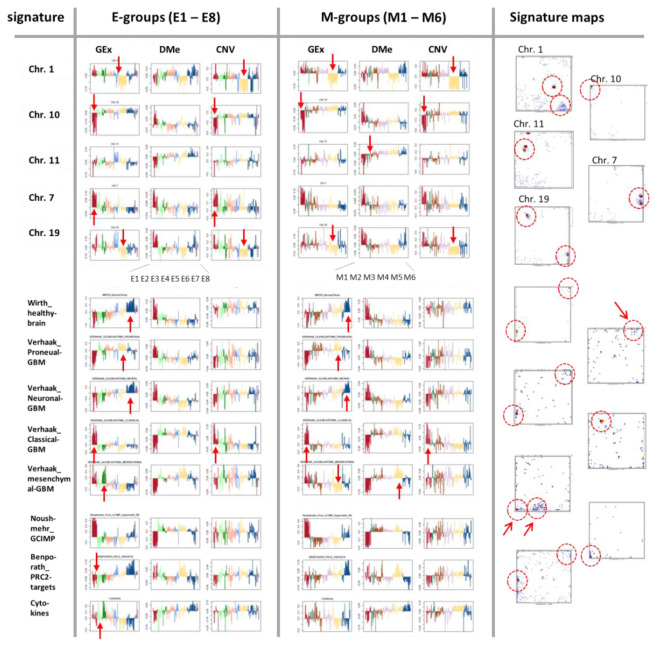
Omics (subtype-sorted) profiles and maps of selected gene signatures. Stratification into eight expression (E-) and six methylation (M-) subtypes show partly sharp subtype-specific differences of feature values not resolved in the genetic groups (see red arrows).

For the largely overlapping groups, the respective omics portraits virtually agree, namely between IDH-wt, E1, and M1 and between IDH-O, E6, and M5. Conversely, IDH-A/A’ split into a series of subtypes, differing in the expression activity of cell cycle as well as inflammatory genes (increase from E4/M4 towards E2,3/M3,2) paralleled by decaying methylation in the respective spots (Figure 7a,b) [35]. Hence, re-stratification of the LGG into the E- and M groups resolves heterogeneity with higher granularity showing partly clear differences, e.g., between the IDH-A/A’ subgroups and between the NL-tumours and the rest (Figure A3).

Profiles of the most pronounced spots illustrate the variations of Gex, Dme and CNV features across the LGG groups and subtypes, e.g., antagonistic overexpression and hypermethylation of IDH-wt which allocates the affected genes in opposite corners (lower left versus upper right) of the map (Figure 7b). This anti-correlation becomes even better resolved in the respective ScoV map (Figure 7c) revealing, e.g., hypermethylation modes in IDH-mut (GCIMP) and IDH-wt (proneural, PN) tumours by blue spots (see [24]). The Gex versus CNV ScoV-profiles indicate direct correlations (red spots) between gene expression and CNV in IDH-wt (E1, M1) in the left upper corner mainly due to Chr10- losses as well as in IDH-O (E6, M5) in the right lower corner due to Chr1/19 codeletions. Comparison of the Gex versus Dme ScoV-profiles, in turn, indicate negative correlations (blue spots) which refer to GCIMP and anti-GCIMP methylation modes (Figure 7c). In summary, resolution of the genetic groups into expression and methylation subtypes further refines the combined omics landscapes, especially of IDH-mut astrocytomas, IDH-A/A’, in terms of functional modes such as alterations of the cell cycle activity and inflammatory response governed by combined changes of methylation and/or CNV modalities. Combined portrayal resolves these coupled modes with high granularity and visualizes them in an intuitive fashion.

### 3.7. Reweighting the Modalities—Single Omics Dominated Maps

**Figure A4:**
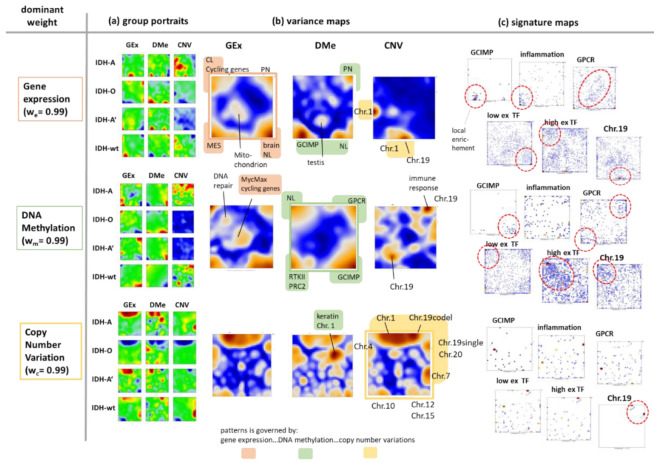
CombiSOM of LGG using dominant weights (w = 0.99) for gene expression, DNA methylation and CNV, respectively. The mean portraits of the genetic groups in the Gex, Dme and CNV domains and the variance maps reflect different topologies of the SOM which are governed by the respective “dominantly weighted” omics domain. They result in different distributions of genes across the maps. (compare with Figure 4a, c referring to the equally weighted combiSOM). (**a**) The mean group portraits enable to identify modules of co-regulated genes of high feature (Gex, Dme, CNV) values in the different genetic groups. (**b**) The variance maps identify spot-regions of co-expressed (Gex map), co-methylated (Dme) and co-aberrant (CNV) genes which partly mutually overlap between the different modalities. (**c**) Distribution of genes in each of the SOM is different. It is governed by the dominantly weighted omics domain giving rise to clusters of highly variant gene expression (Gex), methylation (Dme) or copy numbers. Selected gene signatures consequently distribute differently in the different SOM. GCIMP genes were collected by combined hypermethylation and underexpression [37]. They consequently accumulate in certain areas in the Gex and Dme-dominated maps but diffusely spread over different chromosomal locations in the CNV dominated map. Inflammatory genes [44] show the strongest local enrichment in the Gex dominated SOM but are more distributed in the two other SOM. Genes encoding G-protein coupled receptors (GPCR) accumulate in the middle of the Gex dominated SOM, an area of low variant expression, while in the Dme-dominated SOM these genes associate with higher variance, meaning that to a higher degree with changing methylation than with expression. Low expression transcription factor genes associate more with epigenetic modes of genomics regulation, while high expression TF associate more with transcription factor networks [56].

Our integrative SOM approach combined the Gex, Dme and CNV modalities applying equal weight to each of them (*w*_e_ = *w*_m_ = w_c_ = 1/3; see Figure 1 for illustration). This balanced combiSOM generated a landscape which is affected by all three omics data domains. The alteration of the weight factors under the condition w_e_ + *w*_m_ + *w*_c_ = 1 would increase or to decrease the relative effect of each of the different modalities. In a previous two-modality (Gex and Dme) combiSOM approach on WHO grade IV glioblastoma data, we tuned the weighting factor between dominant weighting of Gex (*w*_e_ = 0.99) and Dme (*w*_m_ = 0.99) [34]. We found that the respective major weight component determines whether gene expression or methylation data show largest variance in the resulting virtually “univariate” SOM. For the LGG data, we here calculated three such weight-dominated combiSOM by setting *w*_e_ = 0.99 (and *w*_m_ = *w*_c_ = 0.005) for the Gex dominant SOM and analogously *w*_m_ = 0.99 or w_c_ = 0.99 for Dme- and CNV dominance, respectively. As the main result, we found that group-averaged portraits and variance maps of the Gex and Dme-dominated SOM show mostly similar topologies governed by the high variant modules of gene expression and DNA methylation, respectively (Figure A4). In contrast, the topology of the CNV dominated SOM is governed by copy number aberrations, e.g., at Chr. 1, 19, 7 and 10 and the respective dose–response relationships between CNV and Gex (Figure A4). In general, tuning the weight factors thus enables tuning the SOM topologies between high Gex variant, high Dme-variant and high CN-variant spot patterns where overlap regions are indicative for couplings between the omics modalities such as repressive interactions between Dme and Gex and dose–response relationships between CNV and Gex. Each of these single-omics dominant SOMs can be used to study these situations in detail. However, each of them provides a separate SOM-topology with different distributions of genes, which requires a new and separate orientation and interpretation of the respective map. Conversely, the equally weighted SOM combines the omics modalities in a balanced fashion, which makes this setting a preferential option for balanced multi-modal omics landscapes. Finally, setting weights in a virtually bi-variant fashion (e.g., *w*_e_ = *w*_m_ = 0.5; *w*_c_ = 0) would generate SOM landscapes governed by the co-variance between two of the omics domains, thus providing a third option for studying relationships between the omes.

## 4. Discussion

### 4.1. Multi-Omics Cartography of LGG

DNA methylation of CpGs in gene promoters, CNV, and gene expression are mutually dependent effects that affect activity of cellular programs. We here presented a method enabling the combined analysis of multi-omics data based on SOM machine learning. It “portrays” the expression, methylation, and CNV landscapes using the same “gene-centred” coordinate system which enables their combined visual evaluation and direct comparison on a personalized and class-related basis for each tumour and subtype, respectively. We applied this combined SOM portrayal to LGG, a relatively well characterized tumour entity which classifies into a series of molecular subtypes defined by genetic key aberrations such as the mutation of the *IDH*-gene and/or co-losses on chromosomes 1 and 19. These changes together cause large-scale effects on DNA methylation and gene expression, and in final consequence, associate with cell fate decisions towards oligodendroglioma- (IDH-O) or astrocytoma- (IDH-A), as well as GBM(IDH-wt)-like tumour cell lineages with different prognostic impact [45].

**Figure 8:**
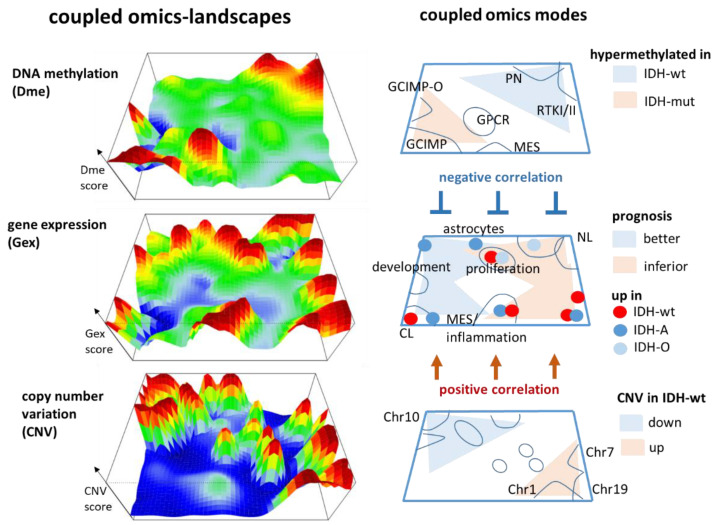
Multi-omics cartography of gliomas. DNA methylation, gene expression, and copy number variations are visualized as three layers of mutually linked landscapes where the (red) peaks refer to variant gene-centred features (DNA promoter methylation, gene expression, and copy number gains, respectively) and (blue) valleys to virtually invariant features. The right part schematically assigns the key topological features and their regulation in LGG.

Our multi-omics cartography visualizes three layers of molecular landscapes which are linked by gene-centred Dme (promoter methylation), Gex (gene expression), and CNV (copy number variation) features (Figure 8). Our method segments the different omics landscapes into modules of co-methylated, co-expressed, and co-aberrant genes which are visualized as peaks in the three dimensional and as spots in the two-dimensional maps, respectively. They reflect the underlying network of regulatory modes of cell activity within each of the omics layers and between them. For example, GCIMP-methylation due to the *IDH* mutation generates such peaks in the left lower and right upper part of the map which hypermethylate in tumours carrying either the mutated or the intact *IDH* gene, respectively. Conversely, key CNV associating with IDH-wt and IDH-O tumours more distribute along the other diagonal from the left upper towards the right lower part of the map. This virtually orthogonal distribution of DNA methylation and genetic CNV effects reflects their partial independence. In contrast, variant transcriptome modules spread throughout the whole map because gene expression is modulated by methylation as well as by CNVs preferentially via repressive and direct interactions, respectively. Certain modules such as the G-protein coupled receptor (GPCR) that peak strongly protrude only in the methylation map, thus illustrating that variations in one of the omes does not necessarily transform into variations in another omics layer (see, e.g., [35] for a detailed discussion). The different modes associate with different functional contexts (Figure 4) and prognoses, which can be visualized in terms of functional and hazard ratio maps, respectively (Figure 5a,b) and which reflect aspects of pathogenesis of LGG.

### 4.2. LGG Pathogenesis Is Governed by Genetic and Epigenetic Factors along Subtype Specific Paths

Particularly, mutations in the *IDH1/2* gene(s) are the driving event behind IDH-mut LGG [35,45]. The resulting misfunction of mutated *IDH1/2* proteins in the TCA cycle induces widespread alterations of DNA methylation via an onco-metabolic mechanism. The aberrant oncometabolite 2-hydroxyglutarate (*2-HG*) inhibits the activity of a series of enzymes erasing or writing methylation marks at the DNA and at histone side chains, which deregulates the epigenetic machinery of the cells, making them more plastic and forming a common progenitor of both IDH-A and IDH-O subtypes. IDH-O gliomas arise after the Chr. 1p/19q co-deletion as genetic hallmark. It deactivates *CIC* (at Chr. 19q), a transcriptional repressor, which in consequence promotes proliferation, blocks differentiation and, in combination with activating mutations in the promoter of the *TERT*-gene, supporting survival of IDH-O tumour cells via a telomerase-driven telomere maintenance mechanism (TEL-TMM). IDH-A, in contrast, lacks the Chr. 1p/19q co-deletion, but instead, its pathogenesis is driven by a triple hit mechanism. First, deactivating mutations of the *TP53* gene (hit 1), a stereotypical tumour suppressor deactivated in more than 50% of all cancers, accumulate excessive mutations and genomics damage in the progressing cells. Second, deactivating mutations of *ATRX* (hit 2), encoding a chromatin remodelling enzyme of the *SWI/SNF* family, alter the chromatin structure in the telomeric regions and promotes cell survival by an alternative telomere maintenance mechanism (ALT-TMM). Third, hit 3 downregulates transcription of *SOX2*, a transcription factor essential for cell differentiation, via a promoter-enhancer dissociation mechanism induced by aberrant local DNA-hypermethylation, which disrupts *CTNF*-induced chromatin looping. In consequence, hit 3 blocks differentiation of the neuronal progenitors. Pathogenesis of IDH-wt gliomas is affected by blocked differentiation driven by key CNV at Chr. 7 and 10 and aberrant function of PRC2 and of its components such as *EZH2*.

Hence, overall pathogenesis of LGG is governed by a complex interplay of genetic and epigenetic mechanisms giving rise to different modes of genomics regulation which associate with key functions such as proliferation, inflammation, and cell differentiation in a subtype specific fashion. Our combiSOM identifies mutual relations between these modes in and between the omics layers. For example, one finds that IDH-wt gliomas selectively share activated proliferation with IDH-O while activated inflammatory features are shared with IDH-A. Increasing inflammatory characteristics of IDH-A associates with decaying GCIMP and overall DNA methylation while, conversely, GPCR methylation increases. Our combiSOM maps directly visualize the mutual association between the Gex and Dme layers, which in independent single omics analytics, are not obvious and required additional analytic efforts for their identification. The prognostic maps link molecular features in all three omics layers with potential impact for molecular marker selection.

### 4.3. What Modality Is the Best?

Our analysis provides intuitive answers to important questions such as “What is the best?”, which is frequently asked about multi-omics settings.

What omics modality is the best? CNV and DNA methylation are “structural” modalities affecting genes with or without explicit functional consequences, e.g., by copy number losses of whole chromosome arms, e.g., of Chr. 10p, 1p or 19q in LGG [22] or by large-scale hypermethylation affecting, e.g., the olfactory subgenome including GPCR as a functional category [25]. These “structural” lesions only partly associate with the transcriptome leading to a lack of one-to-one correspondence as demonstrated previously [34,35]. In consequence, co-methylated or co-aberrant genes selected from the Dme or CNV domains, respectively, usually don’t strongly enrich functional gene signatures due to their “structural” origin which dilutes the functional signature genes often in a “sea” of non-functional ones. However, they can provide footprints of cancerogenesis such as cell of origin characteristics maintained in the Dme patterns [42,46]. Contrarily, co-expressed genes selected from the Gex domain often directly reflect regulatory networks of distinct functional pathways. In consequence, the transcriptome domain seems advantageous for extracting functional information based on the “guilt by association” principle [47] and eventually can be used as filter to extract genes with functional relevance from genetic and methylation patterns.

What modality provides the best markers? Correlation of characteristic genetic markers with clinical outcome defined three major prognostic groups [22] considered in the LGG classification proposed by WHO [48] and here as IDH-A (and IDH-A’), IDH-O, and IDH-wt genetic groups. It stratified patients into prognostically distinct classes better than histological classes. The addition of gene expression markers to this genomics classifier did not further improve prognosis [22]. This result simply shows that due to interactions between the omics modalities their individual prognostic power is partly redundant and their combination doesn’t markedly improve prognosis. However, the HR maps (Figure 5b) reveal that the (red and blue) areas of prognostic impact only partly overlap between the modalities meaning that suited single marker genes from different omics domains can diverge because of their different responsiveness. For example, the *IDH* gene, although probably the most distinctive genetic marker, shows only weak differential expression and methylation between the IDH-mut and IDH-wt groups, while conversely, *CIC* located at Chr. 19 is a suited expression marker to discriminate between IDH-A and IDH-O. Multi-omics cartography in terms of prognostic maps provides a tool to extract such gene signatures of maximum/minimum HR from the different omics domains. Novel genetic and functional markers and signatures for brain tumours are under discussion [49]. Note that other tumour types such as colon cancer [50] or B-cell lymphomas [51] show less clear overall omics landscapes with respect to clinical outcomes which suggests that amendments can be reached presumably by combining different omics modalities.

What subtyping is the best? We independently used expression and methylation data, to refine the genetic groups regarding neuronal-type (NL) LGG of low tumour content distributed over all genetic groups and regarding three to four IDH-A subtypes differing in immunogenic properties partly resembling that observed in pilocytic or mesenchymal astrocytomas [35] with possible impact for treatment resistance and prognosis [25,52]. Multiomics cartography well illustrates subtle differences between the molecular landscapes of the different subtypes (Figure 7a). Expression, methylation, and genetic subtypes largely overlap but show differences in 10–20% of the number of tumours in each of the classes [35]. Consenting subtypes from the different omes would simplify the picture but conversely, would remove important domain-specific details such as methylation of the GPCR genes with possible impact on immunogenicity of the tumour microenvironment. We therefore advocate that omic-specific subtyping should complement consensus classification schemes in order to more specifically considers functional details.

What integration method is the best? Our combiSOM method enables integrating the different domains with variable weights. Equal-weighting combines the omics layers in a balanced fashion, thus revealing mutually integrating features in the gene state space reflecting the inter-omics network of genomics regulation. Tuning the weights towards single-ome dominated landscapes conversely reveals co-variant genes regarding the dominant layer and particularly, non-covariant ones, e.g., if GPCR expression on the average is weakly affected by methylation. We recently used these dominant-omics settings to decipher the cooperation between epigenetics and transcription factor networks for cell fate decisions [53]. 

Hence, the overall answer on all these questions is a Solomonic “it depends” one, where the multi-omics view provides more flexibility and a wider, holistic view in data space for selecting markers and subtypes. More importantly, multi-omics data are mandatory for the comprehensive understanding of the whole repertoire of genomics regulation underlying cancer genesis and development. A similar “Solomonic” conclusion was drawn in a systematic benchmark study comparing multi-omics integration methods on cancer data [54]. The effect of different omics data types varies and can improve the outcome in terms of both clustering and clinical metrics, but it can have even a negative effect if too many omics layers are integrated, thus refuting the intuition that incorporating more types of omics data always helps produce better results. As possible reasons for this counterintuitive observation, the authors see redundancy in the information content of different types of omics data, especially between Gex and Dme data, as well as suboptimal bioinformatics processing in the integration step. These reasons, why one integrative method behaves this way and another in another way, are not clear, mainly because of the “black box” character of the kernel algorithms making it difficult to follow the integral processing step which combines the different domain data. Our integral cartography, conversely, applies a simple weighted combination of the omics domain data together with their intuitive visualization which supports the interpretation of the relatedness between the omics layers and to adjust their combination for the particular question under study.

### 4.4. Limitations and Future Applications

Our publication illustrates the potential of multi-omics SOM cartography. We selected a hitherto well-characterized LGG data set as a worked example for illustration [22,25,35]. Our study is limited by the relatively small sample size of 122 tumours which doesn’t resolve molecular details of part of subtypes. We have previously shown that a larger set of more than 400 LGG available in the TCGA data portal (The Cancer Genome Atlas) well fits into the classification scheme proposed based on our smaller data [25], which supports the reliability of the landscapes presented here. Other limitations are the restriction to methylation of the gene promoter region and to CNV as a specific feature of the genetic domain. We see future applications, for example, by considering explicitly the mutation domain, methylation in the gene body and/or upstream enhancer regions as well as chromatin accessibility as measured by the ATAC (Assay for Transposase-Accessible Chromatin using sequencing) technique often in combination with gene expression data in single-cell settings. Another application of combiSOM addressed layers of different histone modifications obtained by means of ChIP-seq measurements [32].

## 5. Conclusions

Multi-omics SOM cartography allows for disentangling the diversity of regulatory modes of cell functions in terms of easy-to-interpret gene-centric data landscapes. They visualize aberrant changes related to complex diseases such as cancer. The method is not restricted to the triple-omics data used here. It can be extended to integrate other omics features such as genetic mutation or protein expression data. “Phenotype” association with survival of the patients and other clinical characteristics potentially extends the visualization options of the method. Due to the growing use of multi-omics data, we expect that these options will become important for future progress in cancer bioinformatics.

## Data Availability

Gene expression and methylation data are available in the gene expression omnibus (GEO) database under accession number GSE61374 (LGG expression [22]) and GSE129477 (LGG methylation [25]). The R-object is available in the Leipzig Health Atlas under ID 8CUCNFVX6N-6 (see Appendix B).

## Supplementary Materials

The following are available online at https://www.mdpi.com/article/10.3390/cancers14112797/s1, Supplementary File 1: Table S1: Gene lists of spot clusters A–J; Excel Table; File 2: Quick start guide for CombiSOM, PDF.

## Glossary

ATAC	assay for transposase-accessible chromatin
*ATRX*	gene encoding ATP-dependent helicase ATRX, X-linked helicase II
ChIP-Seq	chromatin immunoprecipitation followed by DNA sequencing
Chr	chromosome
CIMP	CpG island methylator phenotype in colorectal cancer
CpG island	genomics regions with high frequency of cytosine and guanine
*CTCF^*	gene encoding 11-zinc finger protein or CCCTC-binding factor
CVN	copy number variation
Dme	DNA methylation
DNA	deoxyribonucleic acid
E1–E8	expression subtypes of LGG
EMT	epithelial-mesenchymal transition
*EZH2*	gene encoding Enhancer Of Zeste 2 Polycomb Repressive Complex 2 Subunit, alias KMT6
GBM	Glioblastoma WHO grade IV
GCIMP	Glioma CpG-Island hyperMethylation Phenotype
GCIMP-O	GCIMP with specific hypermethylation of IDH-O
Gex	gene expression
GO BP	gene sets related to biological processes; part of Gene Ontology database
GPCR	G-protein coupled receptor
GSZ	gene set Z score
2HG	2-hydroxyglutarate, an oncometobolite produced by the mutated IDH enzyme
HR	hazard ratio
IDH	gene encoding isocitrate dehydrogenase
IDH-A	IDH-mutated astrocytoma-like subset of gliomas with chromosome 1p19q intact
IDH-mut	gliomas carrying mutation in IDH genes
IDH-O	IDH-mutated oligodendroglioma-like subset of gliomas with chromosome 1p19q intact
IDH-wt	gliomas with wildtype IDH genes
LGG	lower grade diffuse gliomas (WHO grade II and III)
M1-M6	methylation subtypes of LGG
MES	mesenchymal subtype of glioblastomas according to Verhaak art al. (2010) [55]
*MGMT*	O-6-Methylguanine-DNA Methyltransferase
NL	neuronal-like subset of gliomas according to Verhaak et al. (2010) [55]
PRC2	polycomb repressive complex 2
RTK I/II	GBM methylation class I and II according to Sturm et al. (2012) [41]
ScoV	signed square root covariance
SOM	self-organizing map
*SOX2*	gene encoding sex determining region Y (SRY)- box 2
TCGA	The Cancer Genome Atlas*TERT* telomerase reverse transcriptase
TMM	telomere maintenance mechanisms
WHO	World Health Organization

## Appendix A. CombiSOM Methods Description

### 1. Centralization and Harmonization of the Omics Scores

Gene-centric expression, methylation and copy number data (*E_ns_*, *M_ns_* and C*_ns_*, respectively) were preprocessed as described previously [24] and then centralized by subtracting the respective log-mean averaged over all gliomas as follows.

(A1) $$\Delta e_{ns}={log}_{10}E_{ns}-\frac{1}{S}\sum_{s=1}^{S}{log}_{10}E_{ns}$$

(A2) $$\Delta m_{ns}={log}_{10}M_{ns}-\frac{1}{S}\overset{S}{\underset{s=1}{\sum }}{log}_{10}M_{ns}\;with\;M_{ns}=\frac{\beta_{ns}}{1-\beta_{ns}}$$

(A3) $$\Delta c_{ns}=c_{ns}-\frac{1}{S}\sum_{s=1}^{S}c_{ns}$$

where *n* = 1…*N* is the gene index and *s* = 1…*S* is the sample (tumour) index. For methylation measures, we used “M”-scale (ratio of methylated to unmethylated CpG) instead of beta-scale (fraction of methylated CpG) where methylation was integrated over the promoter region of each gene (from 2000 bp upstream to 200 bp downstream). The centralized expression, methylation and copy number values thus define “omics scores” referring to the differential levels of each gene *n* in the data set studied relative to the respective mean averaged over all tumours. If not stated otherwise, we used the terms over- and underexpression throughout the paper for Δ*e_ns_* > 0 and Δ*e_ns_* < 0, respectively; the terms hyper- and hypo-methylation for Δ*m_ns_* > 0 and Δ*m_ns_* < 0, respectively, and CNV gains and losses for Δ*c_ns_* > 0 and Δ*c_ns_* < 0.

We define “profiles” of the omics scores given as data vector for each gene with the sample-related values as elements:

(A4) $$\Delta e_{n•}=\left(\Delta e_{n1},\ldots ,\Delta e_{nS}\right),\;\;\Delta m_{n•}=\left(\Delta m_{n1},\ldots ,\Delta m_{nS}\right)\;and\;\Delta c_{n•}=\left(\Delta c_{n1},\ldots ,\Delta c_{nS}\right)$$

and “states”, given as data vector for each sample with the gene-related values as elements,

(A5) $$\Delta e_{•s}=\left(\Delta e_{1s},\ldots ,\Delta e_{Ns}\right),\;\Delta m_{•s}=\left(\Delta m_{1s},\ldots ,\Delta m_{Ns}\right)\;and\;\Delta c_{•s}=\left(\Delta c_{1s},\ldots ,\Delta c_{Ns}\right)$$

For combined analysis, data were transformed into a unique, “harmonized” scale by normalizing them with respect to the mean absolute value averaged over all data

(A6) $$\Delta e_{ns}^{*}=\frac{\Delta e_{ns}}{{\left\langle \left|\Delta e\right|\right\rangle }_{all}},\;\Delta m_{ns}^{*}=\frac{\Delta m_{ns}}{{\left\langle \left|\Delta m\right|\right\rangle }_{all}}and\;\Delta c_{ns}^{*}=\frac{\Delta c_{ns}}{{\left\langle \left|\Delta c\right|\right\rangle }_{all}}$$

where $\left\langle \left|\ldots \right|\right\rangle$ denotes averaging of absolute values. This harmonization makes the scales of expression, methylation and CNV data mutually comparable.

### 2. CombiSOM Training

After centralization and harmonization, we merged expression and methylation profiles into combined profiles

(A7) $$\Delta d_{n•}^{*}=\left(w_{e}\cdot \Delta e_{n•}^{*},+w_{m}\cdot \Delta m_{n•}^{*}+w_{c}\cdot \Delta c_{n•}^{*}\right)$$

where the data were combined with different mutual weights chosen from the data intervals *w_e_, w_m_, w_e_ =* (0,1) meeting the condition *w_e_ + w_m_ + w_e_* = 1 (see Figure 1 for a schematic overview of the data processing pipeline). The combined profile vectors are then clustered into so-called metagenes by applying machine learning using Self Organizing Maps (SOM) as implemented in oposSOM [19] in the combiSOM version [34]. The metagenes, in analogy to the input profiles, split into the three metagene vectors of gene expression (Gex), DNA methylation (Dme) and CNV,

(A8) $$\Delta d_{k•}^{meta*}=\left(w_{e}\cdot \Delta e_{k•}^{meta*},w_{m}\cdot \Delta m_{k•}^{meta*},w_{c}\cdot \Delta c_{k•}^{meta*}\right)$$

with *k* = 1,…*K* where *K* is the total number of metagenes. The method uses an iterative training algorithm to ensure that the metagene profiles optimally cover the data space. SOM training is performed in two dimensions by arranging the metagenes in a quadratic grid of size x,y = 1…√*K* used for visualization of the data landscapes in metagene space. We used a SOM of size 45 × 45 and default parametrization of SOM training as implemented in [19].

After SOM training, meta- and single Gex, Dme and CNV data are back transformed into their original scales for visualization and further downstream analysis. Accordingly, each sample studied is characterized by its state of metagene expression, methylation and CNV, respectively. The Gex, Dme and CNV states of each tumour sample were visualized by colour coding the metagene values in the quadratic mosaic grid between red (high scores) to blue (low scores) in maximum–minimum metrics for each sample. This way, one obtains three images per sample which “portrait” its expression, methylation and CNV landscapes separately (Figure 1). Importantly, each metagene is located at the same position in all three maps. It is associated with the same cluster of single genes because of the joint training of Gex, Dme and CNV data. Therefore, all three maps can be directly compared with another, e.g., to identify regions of specific combinations of expression and methylation data.

### 3. Signed Square Root Covariance (ScoV) Maps

Associations between the different omics domains can be estimated by calculating the signed square root covariance (ScoV) between their metagene profiles in a pairwise fashion, e.g., between the Gex and Dme profiles

(A9) $$ScoV_{GEx-DMe}:\;c_{ks}{}^{meta}\left(\Delta e,\Delta m\right)=sign\left(\Delta e_{ks}^{meta}\Delta m_{ks}^{meta}\right)\cdot \sqrt{\left|\Delta e_{ks}^{meta}\Delta m_{ks}^{meta}\right|}$$

which provides the state vector $c_{•s}^{meta}=\left(c_{ls}^{meta},\ldots ,c_{Ks}^{meta}\right)$. It defines the ScoV-landscape of the expression and methylation data of each sample using the SOM-mosaic arrangement and a suited colour code, e.g., red for positive, blue for negative correlations and green for intermediate values about zero. The pairwise combinatorics provides three types of ScoV portraits associating Gex and Dme, GEx and CNV and finally Dme and CNV with

(A10) $$ScoV_{GEx-CNV}:\;c_{ks}{}^{meta}\left(\Delta e,\Delta c\right)\;\mathrm{and}\;ScoV_{CNV-DMe}:\;c_{ks}{}^{meta}\left(\Delta c,\Delta m\right)$$

SOM portraits of the three omics domains and of the three combined ScoV were obtained for each sample. For the sample groups, we calculated mean portraits by averaging the respective metagene values over all individual sample portraits of the respective group.

### 4. The Gene Set Z score (GSZ) and Ternary GSZ-Diagrams

The gene set score (GSZ) estimates the normalized mean log-expression of the member genes of a set in each of the tumour samples. It is calculated as $GSZ_{s}\left(set\right)=\frac{\Delta e_{ns}{}_{n=set}}{SD_{n=set,all\;s}}$, where normalization is provided as Z score by using the standard deviation (SD) of the expression of the set genes in all samples [20,39]. For Dme and CNV data, the GSZ score is calculated analogously by substituting the expression value by the respective methylation and CNV values. For ternary diagrams, GSZ scores were transformed into percentages of the domain values $\%GSZ_{s}\left(set\right)=\frac{GSZ_{s}\left(set\right)’}{\sum GSZ_{s}\left(set\right){’}_{}}$ with $GSZ_{s}\left(set\right)’=GSZ_{s}\left(set\right)-min\left(GSZ_{}\left(set\right)\right)$. $\sum GSZ_{s}\left(set\right)’$ denotes the sum over the Gex, Dme and CNV GSZ’values of the respective tumour samples.

### 5. Tumour Similarity Analysis, Supporting and Prognostic Maps

Similarity analysis compares the SOM portraits of the tumour samples by means of Pearson’s correlation coefficient of their Gex, Dme and CNV values using meta-genes instead of single genes, which has the advantage of improving the representativeness and resolution of the results [20]. The correlation matrix was visualized using pairwise correlation maps (Figure 3a) and correlation net (Figure 3b) representations. The correlation net constructs an unweighted graph by connecting the nodes (samples) whose pairwise correlation coefficient exceeds a given threshold (0.5 here, see [20]).

### 6. Data and Program Availability

The R-workspace of the combiSOM application is available at the Leipzig Health Atlas (LHA) repository under the link https://www.health-atlas.de/data_files/583 (LHA-ID: 8CUCNFVX6N-6, provided 6 May 2022). The quick-start guide is available under https://www.health-atlas.de/presentations/4 (see Supplement File S2).

## Appendix B. Additional Figures and Tables

Particularly, the **Gex governed SOM (w_e_ = 0.99, first row of maps)** reveals a few areas collecting genes of highly variant expression which overlap with variant methylation areas due to the interactions between Dme and Gex. The spot related to cell cycle genes appears virtually only in the Gex map, thus indicating a weak association with Dme (and CNV). Hence, the expression of these genes is only partly affected by differential methylation. Analogously, the most variant areas in the CNV map (caused by Chr. 1/19 codel) only partly match with the expression spots, which indicates that the respective modes are affected not only by CNV.

The **Dme-governed SOM (w_m_ = 0.99, second row of maps)** is driven by differential methylation in four major areas which transform into areas of differential expression due to mostly repressive interactions. Genes related to cell cycling accumulate in the middle of the map. Their expression is driven by transcription factor networks such as *Myc* driven targets and virtually not by methylation and not by CNV.

The **CNV governed SOM (w_C_ = 0.99, third row of maps)** shows a pattern of copy number aberrations which largely directly transforms into the Gex variance map. Compare the “dominantly weighted” maps with the “equally weighted” maps in Figure 4a,c.

**Table A1:** Functional context of expression spots.

Spot	Brief Characteristics	Up/DN	Top Genes ^(a)^	Gene Sets and *p* Value of Enrichment ^(b)^
A	Verhhak CL/MES_UP	IDH-wt, IDH-A/ IDH-O	*INMT, CTHRC1, COL6A3, OAS2, SERPINE, MGP, COL4A2, COL4A1, TNFRSF11, SLC2A10*	WILLSCHER_GBM_Verhaak—CL & MES_up 1 × 10^−9^ HALLMARK_EPITHELIAL_ MESENCHYMAL_TRANSITION 5 × 10^−9^, WU_CELL_MIGRATION -08, Phillips MES up vs. Prolif & PN 3 × 10^−7^
B	GCIMP-meth_UP	IDH-wt/ IDH-A, IDH-O, IDH-A’	*AGAP2, DKK1,* *TRH, DRC1, MEOX2, MMP9, CHIT1, FMOD, DDIT4L, EMILIN3*	Hopp_Sturm_GBM_ IDH_UP 1 × 10^−99^, Noushmehr_ GCIMP_hypermeth 2 × 10^−22^, NOUSHMEHR_GBM_SILENCED_BY_ METHYLATION 5 × 10^−22^
C	healthy_brain	/IDH- A’	*PDYN, PNOC, SLC13A5, TAC1, TBR1, RYR3, SOSTDC1, PTH2R, PVALB, COL23A*	GBM_DN 3 × 10^−77^, WIRTH_Nervous System 1 × 10^−43^, Sturm_ RTK II ‘Classic’_UP_RTK I 2 × 10^−29^, WIRTH_Normal Brain 7 × 10^−32^
D	Chr. 10−	IDH-A, IDH-A’/ IDH-wt	*ARMC3, ITIH2, MCM10, TG, SVIL, IL2RA, MAP3K8, FBXO43, AKR1C2, CCDC3*	Chr 10 1 × 10^−99^, HOPP_Weak_promoter 4 × 10^−13^, Reifenberger_GBM_IDH-wt_DN 1 × 10^−10^
E	Chr. 10−	IDH-A, IDH-O/ IDH-wt	*PLEKHS, SFTPD, AFAP1L2, FAM196A, ADRA2A, ATOH7, DUSP5, ADAMTS1, PRLHR, NKX1-2*	Chr 10 1 × 10^−99^, LASTOWSKA_NEUROBLASTOMA_COPY_ NUMBER_DN 1 × 10^−57^, Reifenberger_GBM_IDH-wt_DN 1 × 10^−32^, ROVERSI_GLIOMA_COPY_NUMBER_DN 1 × 10^−8^
F	Chr.13−	IDH-O/ IDH-A	*POSTN, FAM216B, SOX21, GJB2, KL, SGCG, FREM2, PCID2, CUL4A, SKA3*	Chr 13 1 × 10^−99^
G	Healthy_brain, anti-GCIMP	IDH-O// IDH-wt	*NPAS4, EGR4, CHRM1, MARCH4, OPRK1, GPR83, HS3ST3B, SERTM1, SLC32A1, CALB1*	WIRTH_Nervous System 8 × 10^−76^, GBM_DN 2 × 10^−76^, WILLSCHER_GBM_Verhaak−PN (mut&wt)_up 2 × 10^−60^, Sturm_E5_RTK II ‘Classic’_UP 8 × 10^−49^, Lembcke_TCGA_meth_CIMP.L_UP_CIMP.H_DN 3 × 10^−19^
H	Chr. 7+	IDH-wt/	*HOXA5, SLC13A4, WNT2, DLX5, RARRES2, ELN, AZGP1, HOXA7, EGFR, STEAP1*	Chr 7 1 × 10^−99^, AGUIRRE_PANCREATIC_ CANCER_COPY_NUMBER_UP 8 × 10^−10^
I	Chr. 19−	IDH-A, IDH-wt/ IDH-O, IDH-A’	*NLRP11, DNAAF3, PRKCG, VSIG10L, FOSB, SYT5, ZNF578, NKG7, FPR3, PPP1R3*	Chr 19 1 × 10^−99^, KUUSELO_PANCREATIC_ CANCER_19Q13_AMPLIFICATION 5 × 10^−35^, REACTOME_GENERIC_ TRANSCRIPTION_PATHWAY 4 × 10^−34^, AGUIRRE_PANCREATIC_ CANCER_COPY_NUMBER_UP 4 × 10^−32^, ROVERSI_GLIOMA_COPY_NUMBER_UP 8 × 10^−24^
J	Chr. 1−	IDH-A, IDH-A’, IDH-wt/ IDH-O	*WDR63, SPAG17, C1orf194, C1orf58, VAV3, EPHA2, MFAP2, DMRTA2, SLC7A1, RAD54L*	Chr 1 1 × 10^−99^, HOPP_Heterochrom 1 × 10^−99^, LASTOWSKA_ NEUROBLASTOMA_COPY_NUMBER_DN 1 × 10^−99^, OKAWA_NEUROBLASTOMA_1P36_31_ DELETION 2 × 10^−20^, Weller_LGG_A_vs_O_UP 1 × 10^−8^, Weller_LGG_1p19qDel−vs−intact_DOWN 1 × 10^−8^
K	GBM_Mesenchymal, Inflammation	IDH-wt, IDH-A; IDH-A’/ IDH-O	*CFAP126, COL3A1, C7orf57, METTL7B, CRYBG1, S100A8, CLEC18A, CLEC18C, CLEC18B, CYTL*	Sturm_E4_Mesenchymal_RTK I ‘PDGFRA’_DN 1 × 10^−99^, WILLSCHER_GBM_Verhaak−CL & MES_up 1 × 10^−85^, Lembcke_TCGA−expr_ CIMP.H_UP 5 × 10^−83^, CHEN_METABOLIC_ SYNDROM_NETWORK 2 × 10^−46^, Lembcke_Colonic Inflammation 6 × 10^−46^, Tirosh_Macrophage specific genes−melanoma 3 × 10^−38^, immune system process 1 × 10^−37^

(a) Top 10 genes with largest maximal expression. See Supplementary Table S1 for the full gene list, gene names and further details. (b) Gene sets were implemented in oposSOM ([19,39] and references cited therein). Enrichment was calculated using Fisher’s exact test [57]. Only gene sets with enrichment *p* < 10^−6^ were considered.
