# Supplementary material for: Integrated Multi-Omics Maps of Lower-Grade Gliomas
Source: Cancers (Basel). 2022 Jun 4;14(11):2797. doi: 10.3390/cancers14112797 (PMC9179546; doi:10.3390/cancers14112797)

Multi-omics cartography of glioma heterogeneity

Hans Binder, Maria Schmidt, Lydia Hopp, Arsen Arakelyan, and Henry Loeffler-Wirth

IZBI, Interdisciplinary Centre for Bioinformatics, Leipzig University, Germany

Abstract

Molecular mechanisms of lower grade (II- III) diffuse gliomas (LGG) are still poorly understood, mainly because of their heterogeneity. They split into different subtypes carrying mutations at the IDH gene and into IDH wild type gliomas of glioblastoma-resemblance. For deciphering the interplay between co-expressed and co-methylated genes in high grade gliomas, we recently developed a novel combiSOM portrayal approach which directly combines gene expression and methylation data in a joint machine learning step and which finally enables the joint cartography of both data. This combined SOM data portrayal is not restricted to the two modalities, it can be extended to integrate other omics features to take into account their effect directly. The triple-omics SOM portrayal approach presented here combines patient-matched gene expression, DNA-methylation and copy number variation (CNV) of 121 lower grade glioma (LGG) specimen.

Data portrayal quick start guide

Here we will demonstrate how to generate key data portrayal analyses and visualizations from the LGG combiSOM-workspace available from the Leipzig Health Atlas (https://www.health-atlas.de/publications/964). The workspace was created using the combiSOM package, which can be obtained from Github repository (https://github.com/lydiahopp/multiSOMe).

Loading the workspace

Please download the workspace here (https://www.health-atlas.de/data_files/583) and load it to your R session:

```
load( "LGG combiSOM workspace.RData" )
ls()
```

```
## [1] "env"    "modsom"
```

Generation of single tumor portraits

For each tumor specimen (here id ‘8454H’), three data layers are available in the *metadata* slot, corresponding to the three omic-realms:

```
grep( "8454H", colnames(env$metadata), value=TRUE )
```

```
## [1] "8454H_exp"  "8454H_meth" "8454H_cnv"
```

Each of the three layers can be visualized using standard R functionality
(note that columns of *metadata* need to be converted into a 45x45 matrix according to the SOM topology):

```
par( mfrow=c(1,3), mar=c(1,1,3,1))
image( matrix( env$metadata[,"8454H_exp"], 45 ), col=env$color.palette.portraits(1000),
       axes=FALSE, main="8454H expression" )
image( matrix( env$metadata[,"8454H_meth"], 45 ), col=env$color.palette.portraits(1000),
       axes=FALSE, main="8454H methylation" )
image( matrix( env$metadata[,"8454H_cnv"], 45 ), col=env$color.palette.portraits(1000),
       axes=FALSE, main="8454H CNV" )
```

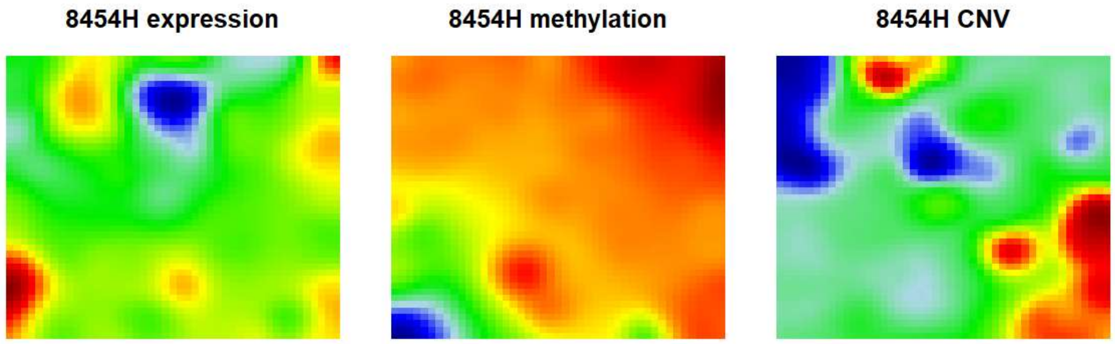

Generation of group portraits

metadata of each omic realm is averaged over all tumor specimen to create a *group.metadata* object:

```
# calculate group.metadata

omic.realm <- sub( ".*_", "", colnames(env$metadata) )
group.metadata <- by( t(env$metadata), paste( env$group.labels, omic.realm ), colMeans )

# output

par( mfcol=c(3,4), mar=c(1,1,3,1))
for( i in 1:length(group.metadata) )
{
  image( matrix( group.metadata[[i]], 45 ), col=env$color.palette.portraits(1000),
         axes=FALSE, main=names(group.metadata)[i] )
}
```

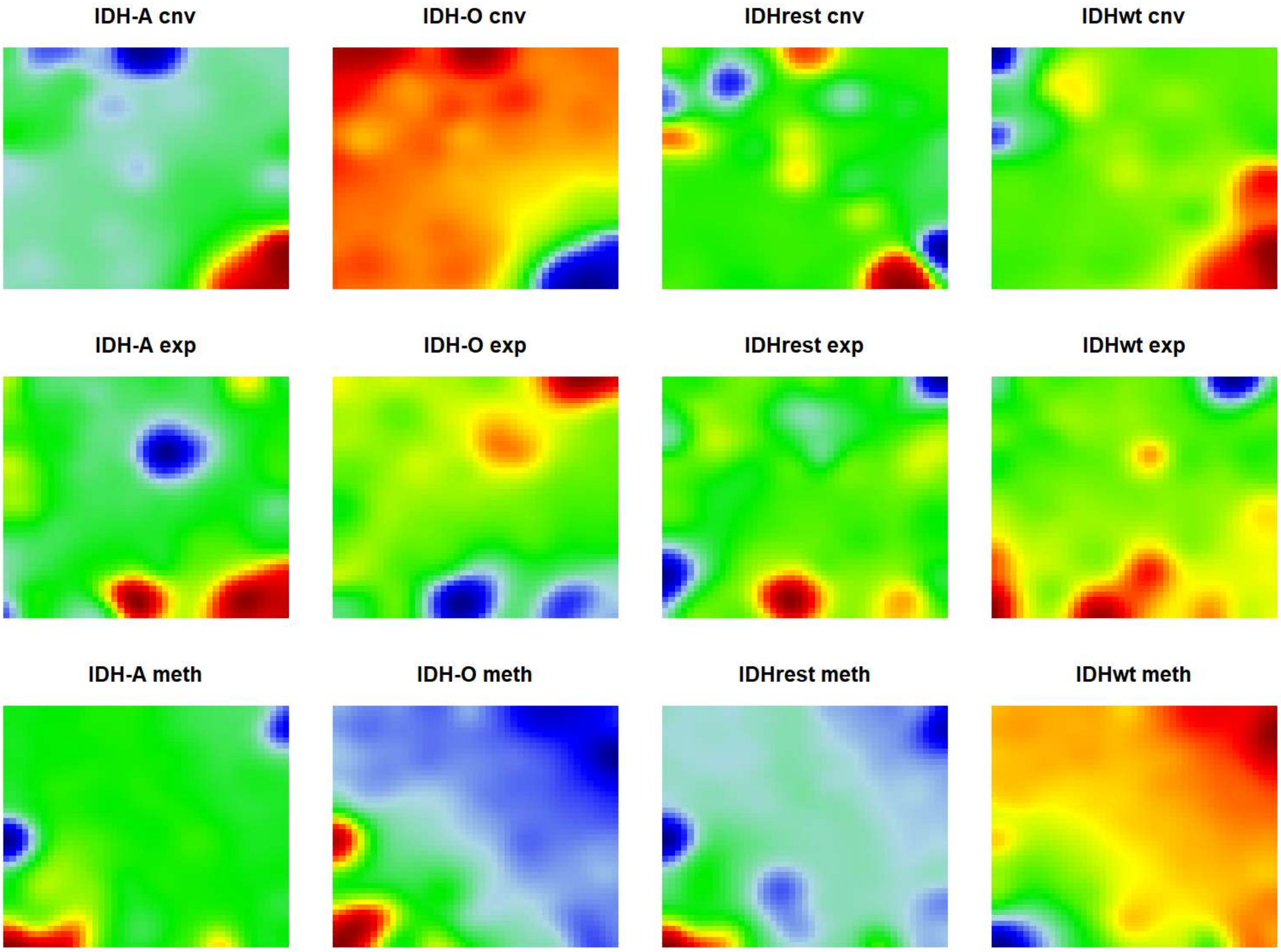

Generation of omic-overview maps

Overview maps are generated as layer-wise maximum of the underlying scaled portraits:

```
# calculate summary maps

minmax.scale <- function(x) { (x - min(x)) / (max(x) - min(x)) }

map.exp <- apply( apply( env$metadata[,which(omic.realm=="exp")], 2, minmax.scale ), 1, max )
map.meth <- apply( apply( env$metadata[,which(omic.realm=="meth")], 2, minmax.scale ), 1, max
)
map.cnv <- apply( apply( env$metadata[,which(omic.realm=="cnv")], 2, minmax.scale ), 1, max )

# output

par( mfrow=c(1,3), mar=c(1,1,3,1))
image( matrix( map.exp, 45 ), col=env$color.palette.portraits(1000),
       axes=FALSE, main="expression summary map" )
image( matrix( map.meth, 45 ), col=env$color.palette.portraits(1000),
       axes=FALSE, main="methylation summary map" )
image( matrix( map.cnv, 45 ), col=env$color.palette.portraits(1000),
       axes=FALSE, main="CNV summary map" )
```

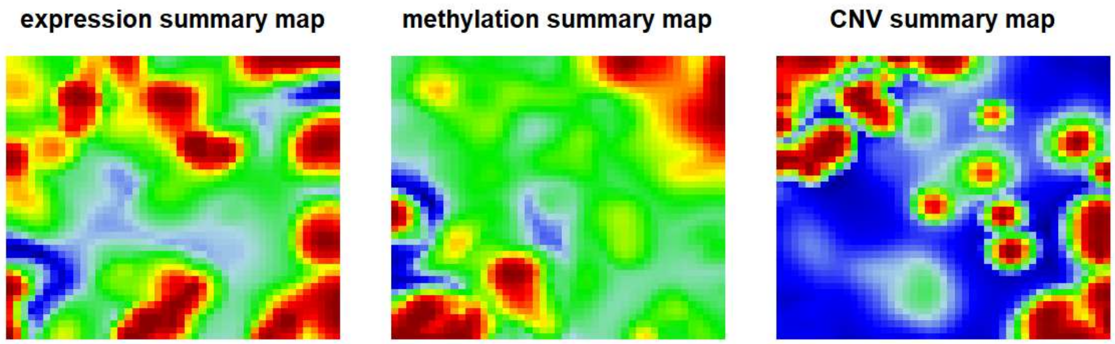

Variance maps are generated as layer-wise variance of the underlying scaled portraits:

```
# calculate variance maps

var.exp <- apply( apply( env$metadata[,which(omic.realm=="exp")], 2, minmax.scale ), 1, var )
var.meth <- apply( apply( env$metadata[,which(omic.realm=="meth")], 2, minmax.scale ), 1, var
)
var.cnv <- apply( apply( env$metadata[,which(omic.realm=="cnv")], 2, minmax.scale ), 1, var )

# output

par( mfrow=c(1,3), mar=c(1,1,3,1))
image( matrix( var.exp, 45 ), col=env$color.palette.portraits(1000),
       axes=FALSE, main="expression variance map" )
image( matrix( var.meth, 45 ), col=env$color.palette.portraits(1000),
       axes=FALSE, main="methylation variance map" )
image( matrix( var.cnv, 45 ), col=env$color.palette.portraits(1000),
       axes=FALSE, main="CNV variance map" )
```

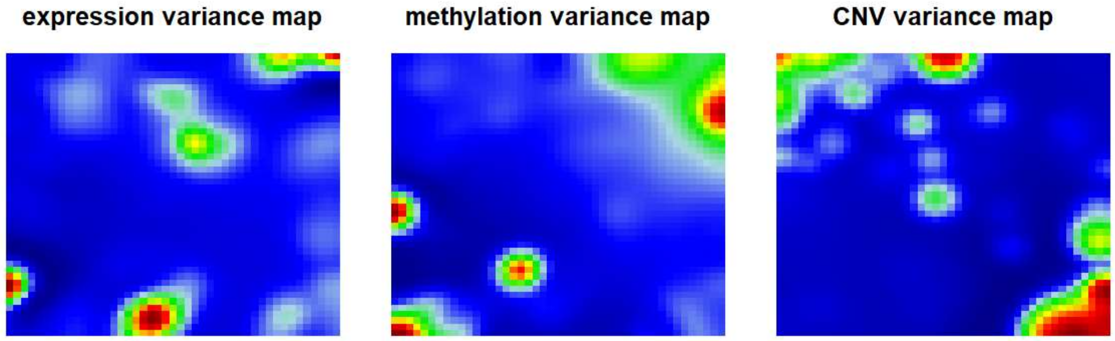

Generation of pairwise correlation maps and correlation networks

Semi-supervised pairwise correlation heatmaps are generated by calculating Pearson's correlation coefficient for each pair of tumors from the same omic-realm, and ordered according to hierarchical clustering within each of the four glioma subtypes:

```
# calculate pairwise correlation maps

pcm.exp <- cor( env$metadata[,which(omic.realm=="exp")] )
pcm.meth <- cor( env$metadata[,which(omic.realm=="meth")] )
pcm.cnv <- cor( env$metadata[,which(omic.realm=="cnv")] )

# intra-subtype hierarchical clustering

order.exp <- unlist( sapply( c("IDH-A","IDH-O","IDHrest","IDHwt"), function(gr)
{
  hc <- hclust( dist( t( pcm.exp[,names( which( env$group.labels==gr & omic.realm=="exp" ))]
 ) ) )
  return( hc$labels[hc$order] )
}))
order.meth <- unlist( sapply( c("IDH-A","IDH-O","IDHrest","IDHwt"), function(gr)
{
  hc <- hclust( dist( t( pcm.meth[,names( which( env$group.labels==gr & omic.realm=="meth"
 ))] ) ) )
  return( hc$labels[hc$order] )
}))
order.cnv <- unlist( sapply( c("IDH-A","IDH-O","IDHrest","IDHwt"), function(gr)
{
  hc <- hclust( dist( t( pcm.cnv[,names( which( env$group.labels==gr & omic.realm=="cnv" ))]
 ) ) )
  return( hc$labels[hc$order] )
}))

# output

heatmap( pcm.exp[order.exp,order.exp], zlim=c(-1,1), Rowv=NA, Colv=NA, col=env$color.palette.
heatmaps(1000),
         labRow=rep("",nrow(pcm.exp)), labCol=rep("",ncol(pcm.exp)), scale="n", main="express
ion PCM",
         ColSideColors=env$group.colors[order.exp], RowSideColors=env$group.colors[order.ex
p])
```

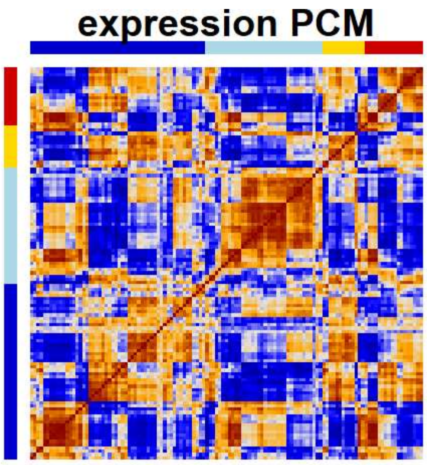

```
heatmap( pcm.meth[order.meth,order.meth], zlim=c(-1,1), Rowv=NA, Colv=NA, col=env$color.palet
te.heatmaps(1000),
         labRow=rep("",nrow(pcm.meth)), labCol=rep("",ncol(pcm.meth)), scale="n", main="methy
lation PCM",
         ColSideColors=env$group.colors[order.meth], RowSideColors=env$group.colors[order.met
h])
```

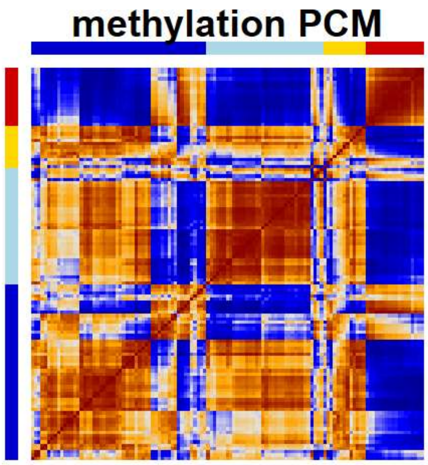


```
heatmap( pcm.cnv[order.cnv,order.cnv], zlim=c(-1,1), Rowv=NA, Colv=NA, col=env$color.palette.
heatmaps(1000),
         labRow=rep("",nrow(pcm.cnv)), labCol=rep("",ncol(pcm.cnv)), scale="n", main="CNV PC
M",
         ColSideColors=env$group.colors[order.cnv], RowSideColors=env$group.colors[order.cn
v])
```

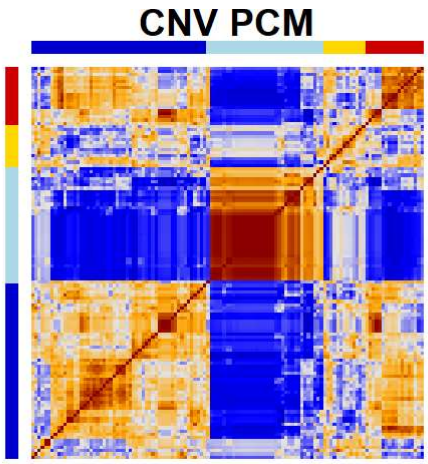

From the pairwise correlation coefficients, a network graph is constructed connecting tumor specimen, represented by the nodes, if their mutual correlation exceeds the threshold 0.5. The *igraph* package is required for this analysis. Note that the layout algorithm is not deterministic:

```
library(igraph)

# calculate adjacency matrices

adj.matrix.exp <- pcm.exp
diag(adj.matrix.exp) <- 0
adj.matrix.exp[ which(adj.matrix.exp < 0.5) ] <- 0

adj.matrix.meth <- pcm.meth
diag(adj.matrix.meth) <- 0
adj.matrix.meth[ which(adj.matrix.meth < 0.5) ] <- 0

adj.matrix.cnv <- pcm.cnv
diag(adj.matrix.cnv) <- 0
adj.matrix.cnv[ which(adj.matrix.cnv < 0.5) ] <- 0

# generate graph objects

g.exp <- graph.adjacency(adj.matrix.exp, weighted=TRUE,  mode="undirected")
g.meth <- graph.adjacency(adj.matrix.meth, weighted=TRUE,  mode="undirected")
g.cnv <- graph.adjacency(adj.matrix.cnv, weighted=TRUE,  mode="undirected")

# output

par( mfrow=c(1,3), mar=c(1,1,3,1))
plot( g.exp, layout=layout_with_kk, vertex.size=8, vertex.label = rep("",ncol(adj.matrix.exp)),
      vertex.color=env$group.colors[which(omic.realm=="exp")], main="Expression correlation network" )
plot( g.meth, layout=layout_with_kk, vertex.size=8, vertex.label = rep("",ncol(adj.matrix.meth)),
      vertex.color=env$group.colors[which(omic.realm=="meth")], main="Methylation correlation network" )
plot( g.cnv, layout=layout_with_kk, vertex.size=8, vertex.label = rep("",ncol(adj.matrix.cnv)),
      vertex.color=env$group.colors[which(omic.realm=="cnv")], main="CNV correlation network" )
```

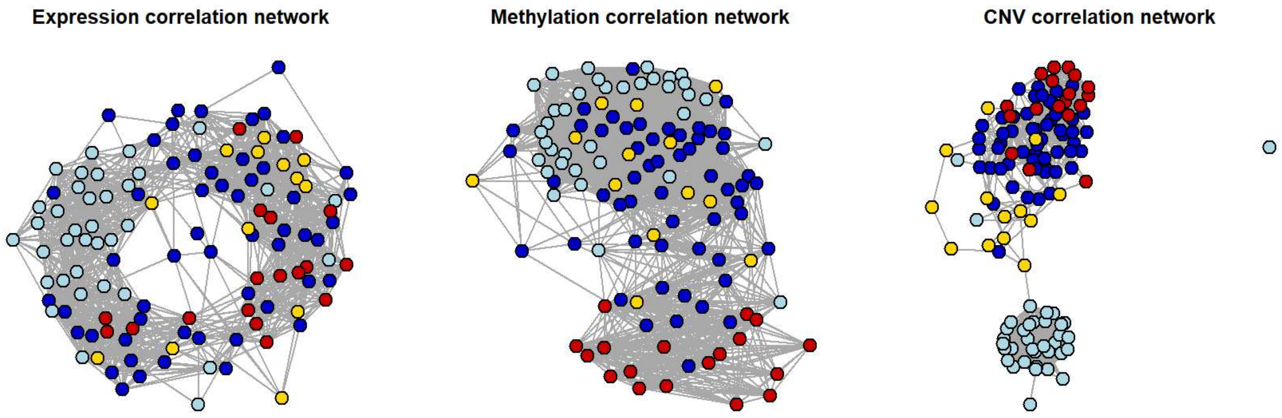

Generation of single feature- and functional enrichment (GSZ-) profiles

Profiles of single feature values and GSZ scores can be extracted from the *indata* and *samples.GSZ.scores* slots:

```
par( mfrow=c(3,1), mar=c(1,3,3,1))

# single gene expression profile

barplot( env$indata[which(env$gene.info$names=="IDH1"), which(omic.realm=="exp") ],
         col=env$group.colors[ which(omic.realm=="exp") ], border = NA,
         names.arg=rep("",sum(omic.realm=="exp")), main="IDH1 expression"  )

# GSZ profiles

barplot( env$samples.GSZ.scores[ "Chr 1", which(omic.realm=="exp") ],
         col=env$group.colors[ which(omic.realm=="exp") ], border = NA,
         names.arg=rep("",sum(omic.realm=="exp")), main="Chromosome 1 expression"  )

barplot( env$samples.GSZ.scores[ "cell cycle", which(omic.realm=="meth") ],
         col=env$group.colors[ which(omic.realm=="meth") ], border = NA,
         names.arg=rep("",sum(omic.realm=="meth")), main="Methylation of cell cycle genes"  )
```

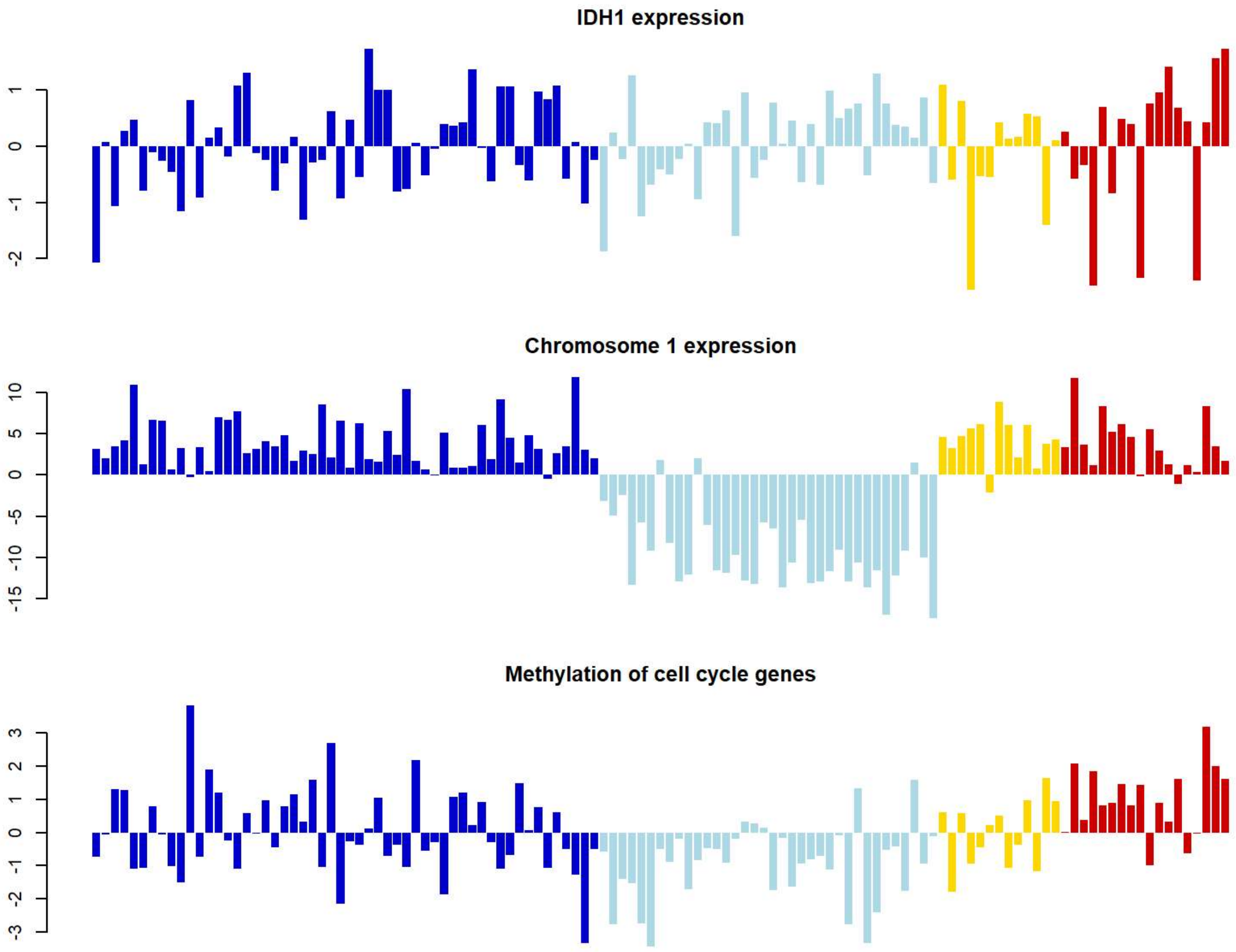

Supplement: Supplementary file 1 [file cancers-14-02797-s001.zip › cancers-1701744 supplementary material/cancers-1701744 supplementary material/Supplement S2-Quick-start-guide.pdf]
